# The Sweden Cancerome Analysis Network - Breast (SCAN-B) Initiative: a large-scale multicenter infrastructure towards implementation of breast cancer genomic analyses in the clinical routine

**DOI:** 10.1186/s13073-015-0131-9

**Published:** 2015-02-02

**Authors:** Lao H Saal, Johan Vallon-Christersson, Jari Häkkinen, Cecilia Hegardt, Dorthe Grabau, Christof Winter, Christian Brueffer, Man-Hung Eric Tang, Christel Reuterswärd, Ralph Schulz, Anna Karlsson, Anna Ehinger, Janne Malina, Jonas Manjer, Martin Malmberg, Christer Larsson, Lisa Rydén, Niklas Loman, Åke Borg

**Affiliations:** Department of Clinical Sciences, Division of Oncology and Pathology, Lund University, Medicon Village 404-A2, SE-22381 Lund, Sweden; Lund University Cancer Center, SE-22381 Lund, Sweden; CREATE Health Strategic Centre for Translational Cancer Research, Lund University, SE-22381 Lund, Sweden; Department of Pathology, Skåne University Hospital, SE-22185 Lund, Sweden; Department of Clinical Sciences, SCIBLU Genomics, Lund University, SE-22381 Lund, Sweden; Department of Pathology and Cytology, Blekinge County Hospital, SE-37185 Karlskrona, Sweden; Department of Pathology, Skåne University Hospital, SE-20502 Malmö, Sweden; Department of Surgery, Lund University and Skåne University Hospital, SE-20502 Malmö, Sweden; Department of Oncology, Skåne University Hospital, SE-22185 Lund, Sweden; Department of Laboratory Medicine, Division of Molecular Pathology, Lund University, SE-22185 Lund, Sweden; Department of Surgery, Lund University and Skåne University Hospital, SE-22185 Lund, Sweden

## Abstract

**Background:**

Breast cancer exhibits significant molecular, pathological, and clinical heterogeneity. Current clinicopathological evaluation is imperfect for predicting outcome, which results in overtreatment for many patients, and for others, leads to death from recurrent disease. Therefore, additional criteria are needed to better personalize care and maximize treatment effectiveness and survival.

**Methods:**

To address these challenges, the Sweden Cancerome Analysis Network - Breast (SCAN-B) consortium was initiated in 2010 as a multicenter prospective study with longsighted aims to analyze breast cancers with next-generation genomic technologies for translational research in a population-based manner and integrated with healthcare; decipher fundamental tumor biology from these analyses; utilize genomic data to develop and validate new clinically-actionable biomarker assays; and establish real-time clinical implementation of molecular diagnostic, prognostic, and predictive tests. In the first phase, we focus on molecular profiling by next-generation RNA-sequencing on the Illumina platform.

**Results:**

In the first 3 years from 30 August 2010 through 31 August 2013, we have consented and enrolled 3,979 patients with primary breast cancer at the seven hospital sites in South Sweden, representing approximately 85% of eligible patients in the catchment area. Preoperative blood samples have been collected for 3,942 (99%) patients and primary tumor specimens collected for 2,929 (74%) patients. Herein we describe the study infrastructure and protocols and present initial proof of concept results from prospective RNA sequencing including tumor molecular subtyping and detection of driver gene mutations. Prospective patient enrollment is ongoing.

**Conclusions:**

We demonstrate that large-scale population-based collection and RNA-sequencing analysis of breast cancer is feasible. The SCAN-B Initiative should significantly reduce the time to discovery, validation, and clinical implementation of novel molecular diagnostic and predictive tests. We welcome the participation of additional comprehensive cancer treatment centers.

**Trial registration:**

ClinicalTrials.gov identifier NCT02306096.

**Electronic supplementary material:**

The online version of this article (doi:10.1186/s13073-015-0131-9) contains supplementary material, which is available to authorized users.

## Background

Breast carcinoma is one of the most common cancers worldwide and a leading cause of cancer-related death in women. Approximately one in nine women will be diagnosed with breast cancer during their lifetime, and in Sweden it accounted for 7,087 new diagnoses and 1,401 deaths in 2011 alone [1]. Contemporary treatment, consisting of surgery, radiotherapy, endocrine therapy, chemotherapy, as well as targeted agents, is driven by standardized clinicopathological criteria and has led to a modest decrease in mortality the last two decades. For example, in the Nordic countries the 5-year survival rate is over 85% [2]. Despite this encouraging statistic, the complete portrait is less than ideal. Unfortunately, approximately 25% of women who survive 5 years will, within the subsequent 15 years, die from recurrent disease [3]. This is in stark contrast to many other cancer types where a 5-year survival is essentially a cure (for example, uterine cancer). On the other hand, it is also recognized that a significant proportion of breast cancer patients are being overtreated: many patients are likely cured by locoregional therapy alone, but are enduring the side effects of unnecessary additional systemic therapies [4]. Our inability to reliably identify such patients has a significant impact on patient quality of life, and adds significantly to the direct economic costs of treating breast cancer as well as the indirect effects on societal productivity [5,6]. Furthermore, we also have limited tools to predict which patients will fail on an indicated therapy due to inherent resistance, or to predict which therapy among statistically equivalent options will be the most effective for an individual patient. Thus, there is still a pressing need for improved biomarkers in breast cancer.

Like all malignancy, breast carcinoma is caused by aberrations in the genome of formerly healthy cells. These aberrations include changes in the normal DNA genetic sequence (for example gene mutations or gains or losses of genetic material) as well as changes in the accessibility and regulation of DNA (such as hypermethylation and chromatin marks). These genomic aberrations affect gene function, and, in concert, also manifest themselves by markedly changing the expression levels of thousands of genes in the tumor from what is the normal pattern in the healthy tissue. Moreover, many of these gene-, genomic-, and gene expression alterations (termed collectively here as biomarkers) are believed to relate to the patient’s prognosis and response to therapy. In breast cancer, the study of gene expression alterations and their relation to clinical outcomes is the most mature, whereas DNA copy number aberrations and clinical course has not advanced as far (with one notable exception, HER2), and much less is understood about somatic mutations and therapy response and survival. Despite much study, there are only a handful of examples of breast cancer biomarkers in clinical use today (for example, the estrogen receptor and HER2).

Recent technological advances have opened exciting new possibilities for studying carcinogenesis at an unprecedented molecular detail, and for developing new clinical tools to improve cancer diagnosis, prognosis, and treatment decision-making. One of the most significant of these new technologies is massively-parallel sequencing, also called next-generation sequencing or deep sequencing [7]. Deep sequencing allows one to ‘read’ the sequence of nucleotide bases of DNA or RNA molecules and identify abnormal sequence variations such as gene mutations and chromosomal rearrangements. Moreover, deep sequencing is also quantitative: the number of sequencing reads that map to a given sequence is proportional to the number of nucleic acid molecules (DNA or RNA) with that sequence in the original sample. Therefore, by sequencing a tumor’s DNA one can measure the DNA copy number of each segment of the genome, and by sequencing a tumor’s messenger RNA (mRNA), one can quantitate the expression level of each gene transcript. In contrast to microarray methods, where expression level or copy number can only be reported for the pre-determined probe sequences that are present on the microarray, an added advantage of deep sequencing is that it operates at the whole-genome scale where a complete representation of the population of DNA or RNA molecules in a sample can be queried simultaneously. Most next-generation technologies are also several orders of magnitude more efficient and less costly than prior sequencing approaches. The cost of gene expression profiling by RNA sequencing (RNA-seq) is similar to the cost of a microarray gene expression experiment. On the other hand, whole-genome sequencing or targeted exome sequencing remains significantly more costly per sample than RNA-seq; however sequencing costs continue to fall. Therefore, routine clinical tests based on tumor deep sequencing can be economically viable, especially considering that many different test results could be reported from a single sequencing analysis.

A major challenge to translating a new cancer biomarker to the clinic is the study sample size. In the development phase, the number of patients studied and its representation of the natural biological and clinical diversity has been inadequate in many studies, usually numbering in only a few hundred samples in the largest studies, and often suffering from various types of selection biases. Validation phase studies often suffer from similar issues. This leads to several consequences, for example the failure to discover potential biomarkers in the development phase, overfitting of data and non-generalizability of biomarkers, and biomarker failure at the validation phase. As a result, thus far there are few multigene assays for breast cancer in limited clinical use: the two most commonly used are a microarray-based test, MammaPrint (Agendia BV), and the OncotypeDX qRT-PCR assay (Genomic Health, Inc). However, these assays are expensive (approximately €3,000 per test), and due to the fact that they were developed based on relatively small study populations (MammaPrint: 78 patients) [8] or only one subgroup of the disease (OncotypeDX: patients with estrogen-receptor-positive tumors with no involved lymph nodes and treated with tamoxifen) [9], the overall clinical utility of these tests beyond the selection of patients with limited benefit from adjuvant chemotherapy, and whether better tests could be developed, has been debated [10,11].

To address these clinical and practical challenges and to continue our efforts to develop improved clinical biomarker tests for breast cancer [12-16], we initiated the multicenter and multidisciplinary consortium, Sweden Cancerome Analysis Network - Breast (SCAN-B) [17] (ClinicalTrials.gov identifier NCT02306096). Launched in the autumn of 2010, the study is fully integrated in the clinical routine and has enrolled more than 6,000 patients to date and collected tumors and blood specimens at a rate of 25 to 30 per week, representing approximately 85% of all breast cancer diagnoses in southern Sweden. Based on our prior experiences, in the first phase we are performing whole-transcriptome RNA-seq and have sequenced over 3,000 breast tumors to date. The primary objectives are to develop, validate, and implement clinically beneficial molecular tumor analyses into the routine healthcare setting for patients with breast cancer in order to improve their care, quality of life, and outcome. Herein we present an overview of the SCAN-B Initiative, our optimized protocols, the status of patient accrual and sample processing for the first 3 years, and the results of initial proof of concept RNA-seq analyses for 49 consecutive patient tumors analyzed in parallel on gene expression microarrays including tumor subtyping and analysis of mutations in cancer-associated genes.

## Methods

### Ethics statement

The study was conducted in accordance with the Declaration of Helsinki and has been approved by the Regional Ethical Review Board of Lund (diary numbers 2007/155, 2009/658, 2009/659, 2014/8), the county governmental biobank center, and the Swedish Data Inspection group (diary number 364-2010). Written information is given by trained health professionals and all patients provided written informed consent.

### Infrastructure

**Figure 1:**
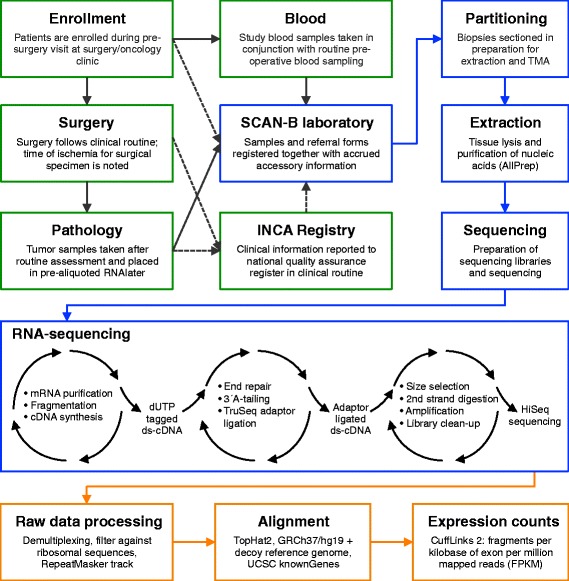
**Overview of the SCAN-B infrastructure.** Shown are the SCAN-B clinical (green boxes), laboratory (blue), and computational and analytical (orange) components. Solid black arrows indicate flow of material, and dashed black lines indicate flow of information. Enrollment and sampling of patients at time of preoperative (neoadjuvant) biopsy is not shown. ds, double-stranded; INCA, Swedish national breast cancer registry; TMA, tissue microarray.

SCAN-B involves researchers, clinicians, and healthcare professionals at Lund University Hospital, Division of Oncology and Pathology, the South Sweden Breast Cancer Group [18], the Regional Cancer Center South, and all seven hospital centers treating breast cancer patients in the Southern Healthcare Region (Malmö, Lund, Helsingborg, Kristianstad, Halmstad, Växjö and Karlskrona), and operates under the auspices of the South Sweden Breast Cancer Group and Regional Cancer Center South. An overview of the study infrastructure is presented in Figure 1.

### Patients and samples

Patient enrollment is integrated and performed as part of the clinical routine (Figure 1). From 30 August 2010, breast cancer patients across the south of Sweden have been offered inclusion in SCAN-B. The eligibility criterion was a preoperative diagnosis of primary invasive breast cancer, and since the autumn of 2012, patients with a preoperative suspicion for breast cancer are also eligible as well as patients receiving neoadjuvant therapy. Patients who participate in SCAN-B receive the same standard of care as patients who do not participate, and at the present time, results from this prospective study are not used to alter any clinical decisions. The study affects the clinical routine minimally. At time of routine preoperative/pre-biopsy blood work, three additional study blood tubes are collected and biobanked as whole blood, buffy coat, plasma, and serum. Clinical routines at surgery, radiology, pathology, and oncology proceed normally. After the routine assessment of the surgical specimen by the pathologist, remainder tumor-cell enriched fresh specimen(s) is placed in a study sample tube(s) containing RNAlater reagent (Ambion) and the time to preservation is recorded. Very small tumors do not always yield excess material for the study, and due to clinical considerations, at present it is difficult to sample cases that appear to be purely or primarily carcinoma *in situ*. For included patients undergoing preoperative biopsy, additional study biopsies are taken and placed in RNAlater. Sample tubes, identified by barcodes, are shipped twice per week at 4°C via inter-hospital transport to the central research laboratory of the Canceromics Branch, Division of Oncology and Pathology, Lund University Cancer Center [19]. Clinical and pathological information tied to the patient and diagnosis as well as follow-up data is retrieved from the national quality registry for cancer patients (INCA) (Figure 1). Postoperative blood samples are also collected as above at 6 months, 12 months, and 36 months after primary surgery. In accordance with ethics and privacy guidelines and laws, clinical and sample information are coded and strictly confidential.

### Tumor sample processing

Tumor specimens sent in RNAlater are processed continuously in our central laboratory (see Additional file 1 for detailed protocols) with handling standards that meet or exceed recommendations of the Breast International Group (BIG). Each tumor specimen is weighed, and when possible, partitioned into three parts: one 30 mg (approximately) piece for simultaneous isolation of DNA, RNA, and protein; one adjacent 10 mg (approximately) piece used for manufacture of a formalin-fixed paraffin-embedded low-density tissue microarray (TMA); and any remainder is stored frozen for future use. The TMA is used for estimation of tumor cellularity and as a research resource. Nucleic acids and protein fraction are isolated from tumor specimen using the AllPrep method and automated using QIAcube machines (Qiagen). RNA and DNA quality control is performed by NanoDrop spectrophotometry and BioAnalyzer (Agilent) or Caliper LabChip XT (PerkinElmer) capillary gel analysis. The extracted RNA, DNA, and flow-through portion that contains proteins and short nucleic acids, are stored frozen for future use. All study information, sampling information, and analysis information are recorded in a secure relational data management and analysis system, BASE [20-22], and user-friendly sample and protocol workflows are interactively generated by the system to ensure standard laboratory operating procedures and efficiency.

### Library preparation for RNA-sequencing

Customized protocols for RNA-seq using 1 μg of starting total RNA were developed and automated for a high-throughput workflow (Figure 1). The complete methods and protocols are described in the Additional file 1. In brief, poly(A) mRNA is isolated from the total RNA in up to 96-well microtiter plate format by two rounds of purification with Dynabeads Oligo (dT)_25_ (Invitrogen) using a KingFisher Flex magnetic particle processor (ThermoScientific). Zinc-mediated fragmentation (Ambion) is performed and the fragmented mRNA retrieved using column purification (Zymo-Spin I-96 plates; Zymo). The sequencing library generation protocol is a modification of the dUTP method, which importantly retains the directionality (stranded-ness) of the sequenced RNA molecules [23,24]. First strand cDNA synthesis is performed using random hexamers and standard dNTP mix followed by cleanup using Sephadex gel filtration (Illustra AutoScreen-96A plates; GE Healthcare), and second strand cDNA synthesis is performed using dUTP in place of dTTP in the dNTP-mix and cleanup using Zymo-Spin I-96 plates. The cDNA is end-repaired and A-tailed, and diluted TruSeq adapters with barcodes are ligated using a modified protocol (Illumina) [23]. Adapter-ligated cDNA is then size-selected to remove short oligonucleotides using carboxylic acid (CA) paramagnetic beads (Invitrogen) and polyethylene glycol (PEG), similar to the previously described methods [25], and automated on the KingFisher Flex. The second cDNA strand is digested using uracil-DNA glycosylase and the product is enriched by 12 PCR cycles (Illumina). The PCR product undergoes two cycles of size selection using CA-beads and varying concentrations of PEG, first to exclude DNA fragments >700 bp and then to exclude fragments <200 bp. Quality control is performed on control libraries using Qubit fluorometric measurement (Life Technologies) and Caliper LabChip XT microcapillary gel electrophoresis. Typically, 10 to 24 barcoded libraries are included in a pool and each pool is sequenced in at least one lane across dual flowcells. Paired-end sequencing of 50 bp read-length is performed on an Illumina HiSeq 2000 instrument.

### RNA-seq gene expression measurements

Raw sequencing read data are demultiplexed using an in-house software and collated by library barcode into sample data sets (Figure 1). Each data set is filtered to remove reads that align (using Bowtie 2 [26] with default parameters except *-k 1 --phred33 --local*) to ribosomal RNA/DNA (GenBank loci NR_023363.1, NR_003285.2, NR_003286.2, NR_003287.2, X12811.1, U13369.1), phiX174 Illumina control (NC_001422.1), and sequences contained in the UCSC hg19 RepeatMasker track (downloaded 14 March 2011). The remaining reads are aligned using TopHat2 [27] to the human genome reference GRCh37/hg19 (with b37 masked chromosome Y and hs37d5 decoy sequences) together with 80,884 transcript annotations from the UCSC knownGenes table (downloaded 10 September 2012). Default TopHat2 parameters are used except for *--mate-inner-dist* (average size with adapters 355, range 268 to 465, measured for each sample individually) *--mate-std-dev 100 --library-type fr-firststrand --keep-fasta-order --no-coverage-search*. Cufflinks v2.1.1 [28] is used to calculate expression levels, fragments per kilobase of exon per million mapped reads (FPKM), using default settings except *--frag-bias-correct --multi-read-correct --library-type fr-firststrand --compatible-hits-norm*. Unmapped reads are processed to be usable by downstream analysis tools using custom software [29]. Read duplication statistics and routine quality assessment were performed using the Bioconductor *Rsamtools* v1.12.4 package [30]. Herein we present analysis for 55 sample libraries generated from 49 tumor specimens, six run with technical replicates using separate aliquots of total RNA. RNA-seq read statistics are presented in Additional file 2: Table S1. Gene expression data were pre-processed by collapsing on 27,979 unique gene symbols (sum of FPKM values of each matching transcript), adding to each gene’s expression measurement 0.1 FPKM, performing a log_2_ transformation, and centering the gene expression values by subtracting the row-wise (gene) median (calculated across the 49 primary data sets) from the values in each row of data.

### Microarray gene expression measurements

To compare to RNA-seq, the same 55 RNA samples as above (49 tumors, six as technical replicates) were analyzed on Human HT12 v4 BeadChip microarrays following the manufacturer’s standard protocol (Illumina). Data from each microarray were pre-processed in BASE [20-22]: background correction was performed, and a constant of 11 was added to each intensity measurement. Genes with missing values in >10% of samples were excluded; otherwise missing values were imputed using k-nearest neighbors implemented in the *impute* R package. The data were quantile normalized using the *preprocessCore* R package, log2 transformed, and each gene was median centered across samples as for the RNA-seq data.

### Molecular subtyping

Intrinsic molecular subtyping was performed by nearest centroid method and Spearman correlation within the *genefu* R package, using three published gene lists (Sørlie, Hu, and PAM50) [31-33]. Mapping of genes between data sets was performed using the *probemapper* 1.0.0 R package [34]. For PAM50, all 50 genes were used for subtyping on both RNA-seq and HT12 platforms; for the Sørlie classification, 432 genes were matched for RNA-seq and 434 genes for HT12; and for Hu classification, 225 genes and 229 genes, respectively. To facilitate unbiased between-platform comparison, each tumor was assigned to the class with the highest correlation. For visualization, hierarchical clustering was performed using the *ConsensusClusterPlus* R package with 1,000 sub-samplings of 80% of samples (or genes; run independently), Pearson distance metric, Ward linkage, and the RNA-seq PAM50 expression values as input. Clusters stabilized at five sample and seven gene clusters.

### RNA-seq mutation analysis

Sequence variants were investigated in known and likely breast cancer driver genes. A list of candidate driver genes of interest was compiled based on the union of genes identified in several large studies: the TCGA breast cancer study (supplementary table 2 in [35]); the Sanger 100 breast cancer exome study (supplementary table 4 in [36]); the Cancer Gene Census of breast cancer drivers [37]; and additional genes with evidence for hereditary breast cancer predisposition. The union resulted in 90 genes (see Additional file 2: Table S2). Using the TopHat-aligned BAM files, pileup files restricted to the exonic regions (plus padding of 10 bases) for these 90 genes were created for each sample using *samtools* v0.1.18 and read metrics were calculated using *bam-readcount* [38]. VarScan v2.3.5 [39] was used to call single nucleotide variants (SNVs) and indels using the following settings: *--min-coverage 2 --min-reads2 2 --min-avg-qual 10 --min-var-freq 0.05 --p-value 1*. The first six bases of each read were ignored for mutation analysis in subsequent steps as mismatches can be introduced by random hexamer priming during library preparation. The local reference sequence around each variant was retrieved using BEDTools [40]. Variant calls were annotated using ANNOVAR [41] with the databases *refGene*, *snp137NonFlagged*, and *cosmic65* from the ANNOVAR website; additional databases of SNVs and indels were created, *tcgaBreast* using data from the Level 2.5.1.0 MAF file from the TCGA Data Portal [42], and *stephens2012* using data from supplementary table 4 in reference [36]. To reduce false positive mutation calls, variants were excluded if they matched any of the following criteria: present in *dbSNP137NonFlagged*, not present in *cosmic65* or *tcgaBreast* or *stephens2012*, located in 5′ or 3′ UTRs, synonymous variants, variants with adjacent homopolymer stretches of ≥5 bases, SNVs with an average base quality of the variant allele <20, and variants with an average distance to the 3′ end of the read <5% of the total read length (after clipping). Thus, only previously identified somatic mutations remained. For plotting amino acid variants, Pfam protein domains were obtained using the *biomaRt* R package by first mapping RefSeq transcript identifiers to UniProt entries within the Ensembl *hsapiens_gene_ensembl* data set, and then querying the InterPro protein data set with these UniProt entries.

### Statistics

Enrollment statistics are based on study records in our relational data management system BASE. The counts for blood samples and tumor specimens are based on the number of patients with at least one sample or specimen collected. To compare the distribution of patient and clinicopathological annotations between sets of patients, Fisher’s exact test was used. A *P* value less than 0.05 was considered significant.

### Bioinformatics implementation

Customized Bash shell scripts, Python, and R code, as well as relevant software packages as described above, were used to perform all bioinformatics analyses.

### Data availability

The RNA-seq and microarray gene expression data herein are available from the NCBI Gene Expression Omnibus [43] under accession GSE60789.

## Results

### Population-based enrollment

**Figure 2:**
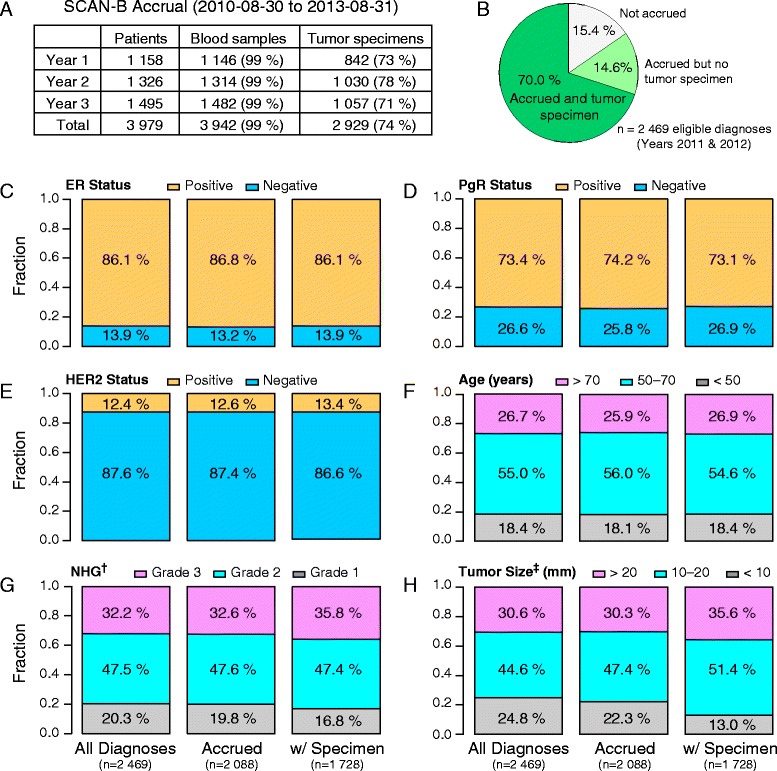
**Study demographics and clinical variables. (A)** For the period 30 August 2010 to 31 August 2013, yearly (non-calendar) summary of the number of enrolled patients, the number of patients with preoperative blood sample collected, and number of patients with tumor specimen collected. **(B-H)** For the two complete calendar years 2011 and 2012 that could be matched to the INCA Swedish national breast cancer registry, **(B)** chart of all cases with a preoperative diagnosis of primary breast cancer within the catchment region divided into those that were accrued or not accrued. Comparison of baseline clinical variables between all eligible breast cancer patients, patients accrued, and patients accrued with tumor sample, for **(C)** estrogen receptor (ER) status, **(D)** progesterone receptor (PgR) status, **(E)** HER2 status, **(F)** age at diagnosis, **(G)** Nottingham histological grade (NHG), and **(H)** tumor size. ^†,‡^ Significant differences were identified between all diagnoses and accrued with tumor specimen for NHG (*P* = 0.005) and tumor size (*P* <0.001), and between patients accrued and accrued with biopsy for NHG (*P* = 0.025) and tumor size (*P* <0.001).

We summarize here the results for the first three years of patient accrual, from 30 August 2010 to 31 August 2013. During this period, 3,961 women and 18 men enrolled in SCAN-B (Figure 2A). For the 2011 and 2012 calendar years, where it was possible to match complete annual records to the Swedish national breast cancer registry (INCA), this represents an estimated 85% of the eligible patient population (with a preoperative diagnosis) within the catchment region (Figure 2B). Approximately 3% of eligible patients decline participation in the study, and 12% are lost to enrollment. There is no bias in terms of clinical variables (estrogen receptor status, progesterone receptor status, HER2 status, patient age, Nottingham grade, or tumor size) between the included patients and the population of all eligible breast cancer diagnoses (Figure 2C-H).

Patients diagnosed with breast cancer are prospectively enrolled at the rate of 25 to 30 per week. For 99% of included patients, preoperative blood samples are biobanked (Figure 2A). For 2,929 patients (74%; Figure 2A), at least one tumor specimen has been submitted to the central laboratory, usually within 1 to 3 days after biopsy/surgery. Most commonly, the reasons for not submitting a tumor specimen include it being judged by the clinical pathologist to be too small for sampling (73%) or the tumor appearing to be carcinoma *in situ* only (7%). The subgroup of patients for which a tumor specimen was collected does not differ significantly from the population of enrolled patients with respect to all clinical variables with the exception of tumor size and Nottingham grade (Figure 2C-H). The median tumor specimen ischemia time, from excision from the patient to placement in preservative solution, is 46 min (interquartile range (IQR), 32 to 65 min), and the median specimen weight is 63 mg (IQR, 34 to 108 mg).

Processing of tumor specimens is performed in near real-time in our central laboratory. As of 31 August 2013, 2,890 of 2,929 tumor specimens (99%) had been partitioned for AllPrep, TMA, and reserve piece, processed, and the nucleic acids isolated (see Methods and Additional file 1). In the first round of processing (with half of the specimen lysate stored frozen for future use), a median of 8.5 μg total RNA (IQR, 3.7 to 16.5 μg) and 15.4 μg DNA (IQR, 7.6 to 25.5 μg) has been isolated per tumor specimen. The isolated nucleic acids are of high purity, with a median 260/280 ratio of 2.05 (IQR, 2.03 to 2.07) and 260/230 ratio 1.93 (IQR, 1.61 to 2.08) for the RNA, and median 260/280 ratio of 1.87 (IQR, 1.86 to 1.88) and 260/230 ratio 1.78 (IQR, 1.38 to 1.99) for the DNA. All unused tissue, lysates, and extracted nucleic acids are stored frozen for future use. The focus of our molecular analyses is initially on whole-transcriptome RNA sequencing using the Illumina HiSeq 2000 platform. For this purpose, greater than 1 μg of total RNA was isolated from 95% of patient samples in the first round of processing, and the median RNA quality score (RQS) is 8.4 (IQR, 7.8 to 8.7).

### RNA sequencing of breast cancer transcriptomes

We have developed a customized high-throughput RNA-seq library generation protocol (Additional file 1). Thus far, it has been used to sequence the transcriptomes of over 3,000 breast tumors. Here we present initial proof of concept results for a representative series of 49 population-based breast cancer patients whose primary surgery occurred during the fall of 2011 and that we analyzed in parallel by RNA-sequencing and gene expression microarrays. From these 49 cases, six were sequenced in technical replicates making for a total of 55 libraries. For each library, a median of 47.6 million passed-filter (PF) paired-reads of 50 bp length were analyzed (IQR, 43.4 to 54.2 million) (Additional file 2: Table S1). An average of 83.0% (range, 73.0% to 88.6%) of the paired-reads remain after initial filtering against a database of non-mRNA targets (passing contamination filter, PCF). Of the remaining reads, a median of 68.0% (IQR, 65.3% to 75.3%) can be mapped to the reference transcriptome map. The average base quality Q-score per read cycle was never below 29, and the duplication rate was low, with a median 63.3% read-pairs being unique (IQR, 55.1 to 69.1%).

**Figure 3:**
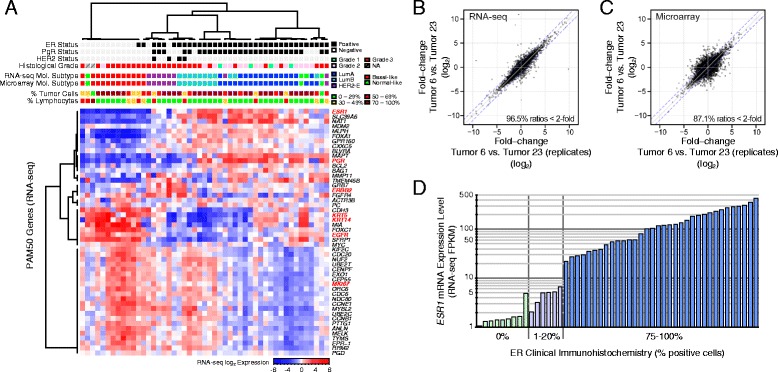
**RNA sequencing and microarray analysis for population-based breast tumors. (A)** Hierarchical clustering of 49 primary breast tumors (clustered columns) using the RNA-seq gene expression measurements and the PAM50 intrinsic gene signature (clustered rows). Clinical annotations for estrogen receptor (ER), progesterone receptor (PgR), and HER2 are indicated below the sample dendrogram, and PAM50 intrinsic subtyping is shown for classification using RNA-seq data as well as using microarray data generated from the same input RNA (90% concordant; results for Sørlie (92%) and Hu (96%) signatures are presented in Additional file 2: Figure S2). Genes of interest are highlighted in red, and relative expression level is indicated by the box color (see color key below the heatmap). For six tumor samples, technical replicates from the same RNA sources were performed for both RNA-seq and microarrays; plotted in **(B)** and **(C)** are representative examples comparing the fold-change for all RefSeq genes between two tumors (Y axis), and the fold-change between the replicated experiments for the same two tumors (X axis). Consistently, RNA-seq demonstrated values closer to the ideal line of identity and for a broader dynamic range. The +/- 2 fold-change (|log_2_| = 1) thresholds are indicated by blue dashed lines. **(D)** RNA-seq-derived expression level of *ESR1*, which encodes the ER alpha protein, is shown compared to the clinical ER IHC score for each of the 49 tumors. See Additional file 2: Figure S3 for corresponding plots for progesterone receptor and *ERBB2* (HER2).

Quantitative gene expression levels, in the form of fragments per kilobase of exon per million mapped reads (FPKM), were derived from the aligned RNA-seq data. As anticipated, the molecular subtypes of breast cancer were readily apparent when classifying tumors using several of the published molecular signatures, such as PAM50 or the intrinsic gene lists of Sørlie *et al.* and Hu *et al.* (Figure 3A and Additional file 2: Figure S2) [31-33]. To compare our RNA-seq method to a prior standard for gene expression profiling, in parallel we performed microarray analysis using Illumina HT12 BeadChips with the same RNA from these 49 tumors, including performing the same six cases in replicate. Concordance of molecular subtypes between RNA-seq and microarray platforms was high using the PAM50, Sørlie, or Hu signatures (90%, 92%, and 96%, respectively) (see also Additional file 2: Figure S2). Gene expression levels derived from RNA-seq compared favorably to microarray-derived gene expression levels. Replicate experiments for six tumors, performed on both platforms, show that the measurement range and reproducibility are higher for RNA-seq, with less apparent noise, as compared to microarrays (Figure 3B and C). The gene expression levels for *ESR1*, which encodes the estrogen receptor alpha (ER) receptor, was compared to the clinical ER immunohistochemistry scores and illustrates the wide dynamic range of RNA-seq for an important breast cancer biomarker (Figure 3D). Corresponding plots are shown for *PGR* (progesterone receptor (PgR)) and *ERBB2* (HER2) in Additional file 2: Figure S3.

### Mutation screening by RNA sequencing

**Figure 4:**
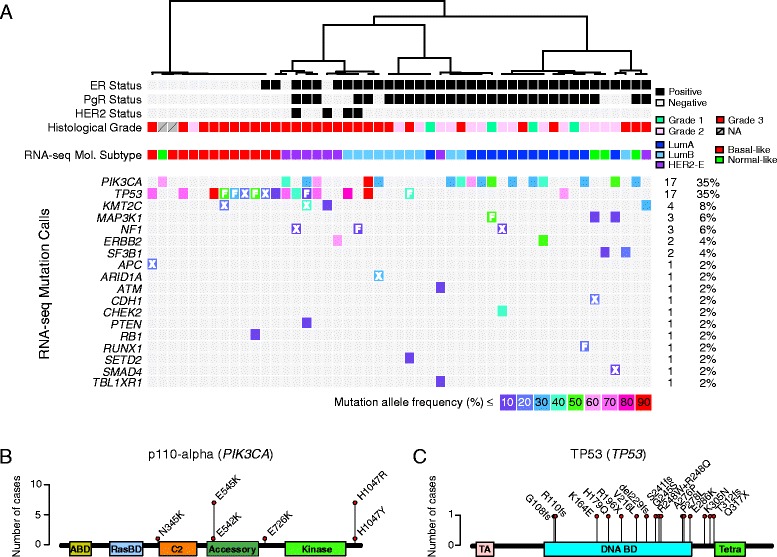
**Detection of mutations by RNA-seq. (A)** Eighteen genes with at least one mutation (out of 90 genes screened) across the 49 population primary breast tumors are shown, in order of frequency (see totals and percentages to the right of each gene row). Mutant allele frequency is indicated by the box color (see key below matrix). All mutations are non-synonymous missense mutations except those indicated by F (frameshift) and X (nonsense). Tumor sample dendrogram is as in Figure 3A. Predicted mutant amino acids are shown for **(B)** *PIK3CA* which encodes the p110-alpha catalytic subunit of the phosphatidylinositol-4,5-bisphosphate 3-kinase oncogene, and **(C)** *TP53* which encodes the tumor suppressor TP53.

In addition to these technical attributes, RNA-seq data can be used to detect gene mutations, splice variants, and fusion transcripts, opening up new avenues of study. As proof of principle, we utilized the 49 tumor RNA-seq data to screen for sequence alterations in 90 genes known to be mutated in human breast cancers (Figure 4A; Additional file 2: Table S2; GSE60789). Typical mutations were detected in these breast cancers with the expected frequencies and association to clinicopathological characteristics: for example, 17/49 (35%) cases were determined to harbor mutations in the oncogene *PIK3CA* and these occurred most frequently in luminal A (8/14), HER2-enriched (3/8), and normal-like (2/4) tumors [35,44]. Similarly, *TP53* was found to be mutated in 17/49 (35%), almost exclusively in grade 3 tumors (16/17), most frequently within the basal-like (9/12), HER2-enriched (4/8) and luminal B (3/11) subtypes, and least frequently in normal-like (0/4) and luminal A tumors (1/14). The detected spectrum of mutations was in-line with expectations: for example, *PIK3CA* mutations affecting residue H1047 in the kinase domain of p110-alpha protein were the most frequently observed, whereas none of the *TP53* mutations were observed more than once in this series of population-based cases (Figure 4B and C). Mutations in 18 out of 90 genes investigated could also be reliably detected in the transcriptome, such as *KMT2C* (*MLL3*), *MAP3K1*, *ERBB2*, *ARID1A*, *PTEN*, and *RB1*, and 39/49 (80%) of tumors had at least one of these 18 genes mutated (Figure 4A).

Tissue microarrays, constructed from a piece of each tumor adjacent to that used for nucleic acid extractions, were evaluated for cellularity composition by hematoxylin and eosin staining and scored for invasive tumor, *in situ* tumor, normal epithelium, lymphocyte, stroma, and adipocyte content. Generally, approximately 75% of cases contain >50% tumor cells and 15% of cases contain less than 30% tumor cells. Few cases appear to be overtly affected by tumor cell content with respect to supervised analyses, for example in the intrinsic molecular subtyping or mutation analysis (Figures 3A and 4A).

## Discussion

We have developed a mature infrastructure for prospective, multicenter, population-based, enrollment of breast cancer patients, coupled to an optimized genomics platform for gene expression profiling and mutation analysis by RNA sequencing. Powerful biomarker discovery projects will be possible after we have studied many hundreds to many thousands of breast tumors and related these data to patient characteristics, treatment response, and outcomes. The established infrastructure will enable SCAN-B-derived biomarker tests to be validated using independent series of population-based cases from the ongoing prospective SCAN-B study. In a similar way, biomarker tests (such as gene expression signatures) from the literature can be tested and validated within our patient material. For validated biomarker tests that are proven to be clinically relevant, the goal is to perform the analysis as a diagnostic test and communicate the result back to the treating physicians within a clinically-actionable time-frame (within weeks after surgery or biopsy). Thus, within the framework of an initiative such as SCAN-B, the cycle time from biomarker discovery, to independent validation, to clinical implementation can be made more rapid and efficient.

With the current participating sites, the SCAN-B Initiative has and will continue to assemble a very large series of breast cancer cases over many years, prospectively analyzed with the same methods and platforms. The first phase of SCAN-B prioritizes the sequencing of expressed mRNAs because of our prior experience and interest, the maturity of the field, experimental cost, as well as the fact that expression level as well as isoform and variant status can be ascertained simultaneously. The wealth of small and long non-coding RNAs, DNA-level aberrations, and epigenetic changes are not yet investigated. Future analyses will investigate global mutational portraits and differential expression of gene isoforms, and ample study material is stored for future genomic, transcriptomic, and proteomic analyses such as whole-genome and targeted exome sequencing, sequencing of non-coding RNAs, and studies of active proteins. The SCAN-B Initiative will enable numerous types of investigations that are population-based and appropriately powered. For example, we aim to identify and validate RNA and DNA biomarkers predicting exceptionally favorable prognosis without need for adjuvant therapy, biomarkers for resistance to specific therapies, such as trastuzumab resistance or resistance to endocrine therapy, and biomarkers to refine the intermediate prognosis cases, such as tumors of histological grade 2. Within the coming years, we will have amassed many cohorts of hundreds to thousands of patients receiving any particular standard treatment, linked to >5 years follow-up history, and with corresponding RNA-sequencing data. Gene expression patterns and mutational patterns, and other biologically relevant information discernible from the SCAN-B data such as expression of alternatively spliced transcripts, fusion genes, and allele-specific expression, will be analyzed in the context of the clinicopathological information, therapy, and patient outcome in order to develop and validate new biomarker tests for eventual clinical use. We also aim to use the SCAN-B infrastructure to identify patients who may benefit from participation in specific clinical trials, for example to select patients whose gene expression or gene mutation status suggests sensitivity to an emergent therapeutic.

Tumor tissues, grossly dissected at pathology, are the most practical samples to analyze in a large-scale setting as compared to microdissection; moreover, the non-tumoral gene expression signals, such as from immune cells and stroma, may be highly biologically and clinically relevant. For example, it has been shown that immune response signatures can be predictive of outcome across a wide range of cancer forms [45-48]. Therefore, depending on the purpose, we foresee the importance of interpreting genomic biomarker results in the context of the estimated compartmental cellularity of each analyzed specimen.

Comparison of mutation analysis at the level of mRNA- versus DNA-sequence warrants further investigation and is currently underway. Based on our early experiences as well as the work of others, we posit that mutations of oncogenes should be efficiently detectable by RNA-seq, but that some mutations in tumor suppressor genes may be more difficult to detect due to lowered expression levels, loss of heterozygosity, and/or nonsense mediated decay [49-51]. We hope to add DNA-level profiling to the SCAN-B routine in the future. For example, the BRCAsearch subproject is investigating, after additional informed consent, the consecutive testing of germline BRCA mutations. Another project currently in progress is comparing the classification of the five conventional clinical biomarkers (clinical determinations for ER, PgR, HER2, Ki67, and histological grade) to paired classifications based on RNA-seq gene expression signatures. The influence of intra-tumoral heterogeneity, subclonality, other cell types such as non-tumoral epithelial and stromal cells, and the limitations of sampling require further study. For patients receiving primary medical treatment, we are currently implementing an extensive sampling program including sequential blood sampling and an additional tumor biopsy after two cycles of preoperative chemotherapy. We also plan to soon begin systematic collection and analysis of metastatic breast cancer samples upon disease relapse, which will provide a rich and informative platform for studying tumor progression and tumor evolution. We believe these results and future results from SCAN-B will complement the existing clinical and pathological evaluation and can become another part of our armamentarium in diagnosing, evaluating, and treating breast cancer.

We present the feasibility of large-scale multicenter collection of population-based breast cancer patient material and analysis with next-generation genomic analytical methods. In our hands, 85% of new diagnoses are enrolled across a wide geography of Sweden, and the specimen collection reflects well the clinicopathological characteristics of breast cancer in the catchment region. Due to primacy of the clinical diagnostic evaluation, very small/low-grade tumors are slightly under-sampled. We anticipate improvements in patient enrollment, and increases in the fraction where a tumor specimen can be collected, as the procedures become further integrated into the healthcare routine and the importance of tissue and blood sampling for genomic analyses becomes further evident through forthcoming studies from us and others. We are expanding the infrastructure to include patients diagnosed with metastatic breast cancer, and also drawing blood samples at routine intervals during the clinical course for liquid biopsy studies.

Lastly, we extend an open invitation to other hospital systems in Sweden and the Nordic countries to join the SCAN-B network. Most recently, Uppsala County joined the network in October 2013. SCAN-B may also serve as a model for similar translational projects in other types of cancer and diseases.

## Conclusions

In summary, we present the successful implementation of a multicenter infrastructure for genomic biomarker development in breast cancer across a wide geography of Sweden, and the optimization of RNA-seq protocols for high-throughput analyses. To our knowledge, this is the largest endeavor of its kind and is distinctive in its population-based approach.

## Additional files

Additional file 1: Figure S1. Materials and Methods. Additional file 2: Figure S2. Figure S3, Table S1, and Table S2.

## References

[1] Engholm G, Ferlay J, Christensen N, Bray F, Gjerstorff ML, Klint A (2010). NORDCAN–a Nordic tool for cancer information, planning, quality control and research. Acta Oncol.

[2] Coleman MP, Forman D, Bryant H, Butler J, Rachet B, Maringe C (2011). Cancer survival in Australia, Canada, Denmark, Norway, Sweden, and the UK, 1995-2007 (the International Cancer Benchmarking Partnership): an analysis of population-based cancer registry data. Lancet.

[3] Brenner H, Hakulinen T (2002). Very-long-term survival rates of patients with cancer. J Clin Oncol.

[4] Dodwell D, Thorpe H, Coleman R (2009). Refining systemic therapy for early breast cancer: difficulties with subtraction. Lancet Oncol.

[5] Gordon L, Scuffham P, Hayes S, Newman B (2007). Exploring the economic impact of breast cancers during the 18 months following diagnosis. Psychooncology.

[6] Armstrong K (2012). Can genomics bend the cost curve?. JAMA.

[7] Meyerson M, Gabriel S, Getz G (2010). Advances in understanding cancer genomes through second-generation sequencing. Nat Rev Genet.

[8] Van’t Veer LJ, Dai H, van de Vijver MJ, He YD, Hart AA, Mao M (2002). Gene expression profiling predicts clinical outcome of breast cancer. Nature.

[9] Paik S, Shak S, Tang G, Kim C, Baker J, Cronin M (2004). A multigene assay to predict recurrence of tamoxifen-treated, node-negative breast cancer. N Engl J Med.

[10] Ahmed AA, Brenton JD (2005). Microarrays and breast cancer clinical studies: forgetting what we have not yet learnt. Breast Cancer Res.

[11] Reis-Filho JS, Westbury C, Pierga JY (2006). The impact of expression profiling on prognostic and predictive testing in breast cancer. J Clin Pathol.

[12] Hedenfalk I, Duggan D, Chen Y, Radmacher M, Bittner M, Simon R (2001). Gene-expression profiles in hereditary breast cancer. N Engl J Med.

[13] Gruvberger S, Ringner M, Chen Y, Panavally S, Saal LH, Borg A (2001). Estrogen receptor status in breast cancer is associated with remarkably distinct gene expression patterns. Cancer Res.

[14] Saal LH, Johansson P, Holm K, Gruvberger-Saal SK, She QB, Maurer M (2007). Poor prognosis in carcinoma is associated with a gene expression signature of aberrant PTEN tumor suppressor pathway activity. Proc Natl Acad Sci U S A.

[15] Staaf J, Ringner M, Vallon-Christersson J, Jonsson G, Bendahl PO, Holm K (2010). Identification of subtypes in human epidermal growth factor receptor 2–positive breast cancer reveals a gene signature prognostic of outcome. J Clin Oncol.

[16] Jonsson G, Staaf J, Vallon-Christersson J, Ringner M, Gruvberger-Saal SK, Saal LH (2012). The retinoblastoma gene undergoes rearrangements in BRCA1-deficient basal-like breast cancer. Cancer Res.

[17] Sweden Cancerome Analysis Network - Breast. Available at: http://scan.bmc.lu.se/.

[18] Alkner S, Bendahl PO, Ferno M, Manjer J, Ryden L (2011). Prediction of outcome after diagnosis of metachronous contralateral breast cancer. BMC Cancer.

[19] Lund University. Faculty of Medicine - Oncology and Pathology. Available at: http://www.med.lu.se/canceromics.

[20] Saal LH, Troein C, Vallon-Christersson J, Gruvberger S, Borg A, Peterson C. BioArray Software Environment (BASE): a platform for comprehensive management and analysis of microarray data. Genome Biol. 2002;3:SOFTWARE0003. 10.1186/gb-2002-3-8-software0003 PMC139402 12186655

[21] Troein C, Vallon-Christersson J, Saal LH (2006). An introduction to BioArray Software Environment. Methods Enzymol.

[22] Vallon-Christersson J, Nordborg N, Svensson M, Hakkinen J (2009). BASE–2nd generation software for microarray data management and analysis. BMC Bioinformatics.

[23] Parkhomchuk D, Borodina T, Amstislavskiy V, Banaru M, Hallen L, Krobitsch S (2009). Transcriptome analysis by strand-specific sequencing of complementary DNA. Nucleic Acids Res.

[24] Nalpas NC, Park SD, Magee DA, Taraktsoglou M, Browne JA, Conlon KM (2013). Whole-transcriptome, high-throughput RNA sequence analysis of the bovine macrophage response to Mycobacterium bovis infection in vitro. BMC Genomics.

[25] Borgstrom E, Lundin S, Lundeberg J (2011). Large scale library generation for high throughput sequencing. PLoS One.

[26] Langmead B, Salzberg SL (2012). Fast gapped-read alignment with Bowtie 2. Nat Methods.

[27] Kim D, Pertea G, Trapnell C, Pimentel H, Kelley R, Salzberg SL (2013). TopHat2: accurate alignment of transcriptomes in the presence of insertions, deletions and gene fusions. Genome Biol.

[28] Trapnell C, Roberts A, Goff L, Pertea G, Kim D, Kelley DR (2012). Differential gene and transcript expression analysis of RNA-seq experiments with TopHat and Cufflinks. Nat Protoc.

[29] Brueffer C. TopHat Recondition. Python script. Available at: https://github.com/cbrueffer/tophat-recondition.

[30] Morgan M, Pages H. Rsamtools: Binary alignment (BAM), variant call (BCF), or tabix file import. R package version 1.12.4. Available at: http://www.bioconductor.org/packages/release/bioc/html/Rsamtools.html.

[31] Sørlie T, Tibshirani R, Parker J, Hastie T, Marron JS, Nobel A (2003). Repeated observation of breast tumor subtypes in independent gene expression data sets. Proc Natl Acad Sci U S A.

[32] Hu Z, Fan C, Oh DS, Marron JS, He X, Qaqish BF (2006). The molecular portraits of breast tumors are conserved across microarray platforms. BMC Genomics.

[33] Parker JS, Mullins M, Cheang MC, Leung S, Voduc D, Vickery T (2009). Supervised risk predictor of breast cancer based on intrinsic subtypes. J Clin Oncol.

[34] Allen JD, Wang S, Chen M, Girard L, Minna JD, Xie Y (2012). Probe mapping across multiple microarray platforms. Brief Bioinform.

[35] Atlas TCG (2012). Comprehensive molecular portraits of human breast tumours. Nature.

[36] Stephens PJ, Tarpey PS, Davies H, Van Loo P, Greenman C, Wedge DC (2012). The landscape of cancer genes and mutational processes in breast cancer. Nature.

[37] Futreal PA, Coin L, Marshall M, Down T, Hubbard T, Wooster R (2004). A census of human cancer genes. Nat Rev Cancer.

[38] bam-readcount. Available at: https://github.com/genome/bam-readcount/.

[39] Koboldt DC, Zhang Q, Larson DE, Shen D, McLellan MD, Lin L (2012). VarScan 2: somatic mutation and copy number alteration discovery in cancer by exome sequencing. Genome Res.

[40] Quinlan AR, Hall IM (2010). BEDTools: a flexible suite of utilities for comparing genomic features. Bioinformatics.

[41] Wang K, Li M, Hakonarson H (2010). ANNOVAR: functional annotation of genetic variants from high-throughput sequencing data. Nucleic Acids Res.

[42] The Cancer Genome Atlas. Available at: https://tcga-data.nci.nih.gov/.

[43] Gene Expression Omnibus. Available at: http://www.ncbi.nlm.nih.gov/geo/.

[44] Saal LH, Holm K, Maurer M, Memeo L, Su T, Wang X (2005). PIK3CA mutations correlate with hormone receptors, node metastasis, and ERBB2, and are mutually exclusive with PTEN loss in human breast carcinoma. Cancer Res.

[45] Budhu A, Forgues M, Ye QH, Jia HL, He P, Zanetti KA (2006). Prediction of venous metastases, recurrence, and prognosis in hepatocellular carcinoma based on a unique immune response signature of the liver microenvironment. Cancer Cell.

[46] Jais JP, Haioun C, Molina TJ, Rickman DS, de Reynies A, Berger F (2008). The expression of 16 genes related to the cell of origin and immune response predicts survival in elderly patients with diffuse large B-cell lymphoma treated with CHOP and rituximab. Leukemia.

[47] Roepman P, Jassem J, Smit EF, Muley T, Niklinski J, van de Velde T (2009). An immune response enriched 72-gene prognostic profile for early-stage non-small-cell lung cancer. Clin Cancer Res.

[48] Criscitiello C, Azim Jr HA, Schouten PC, Linn SC, Sotiriou C. Understanding the biology of triple-negative breast cancer. Ann Oncol. 2012;23:vi13–18. 10.1093/annonc/mds188 23012296

[49] Shah SP, Roth A, Goya R, Oloumi A, Ha G, Zhao Y (2012). The clonal and mutational evolution spectrum of primary triple-negative breast cancers. Nature.

[50] Tang X, Baheti S, Shameer K, Thompson KJ, Wills Q, Niu N (2014). The eSNV-detect: a computational system to identify expressed single nucleotide variants from transcriptome sequencing data. Nucleic Acids Res.

[51] Wilkerson MD, Cabanski CR, Sun W, Hoadley KA, Walter V, Mose LE (2014). Integrated RNA and DNA sequencing improves mutation detection in low purity tumors. Nucleic Acids Res.

