# Supplementary material for: The Sweden Cancerome Analysis Network - Breast (SCAN-B) Initiative: a large-scale multicenter infrastructure towards implementation of breast cancer genomic analyses in the clinical routine
Source: Genome Med. 2015 Feb 2;7(1):20. doi: 10.1186/s13073-015-0131-9 (PMC4341872; doi:10.1186/s13073-015-0131-9)
Supplement: Additional file 1: Figure S1. — Materials and Methods. [file 13073_2015_131_MOESM1_ESM.pdf]

The Sweden Canceromics Analysis Network – Breast (SCAN-B) Initiative: a large-scale multicenter infrastructure towards implementation of breast cancer genomic analyses in the clinical routine

ADDITIONAL FILE 1

Lao H. Saal, Johan Vallon-Christersson, Jari Häkkinen, Cecilia Hegardt, Dorthe Grabau, Christof Winter, Christian Brueffer, Man-Hung Eric Tang, Christel Reuterswärd, Ralph Schulz, Anna Karlsson, Anna Ehinger, Janne Malina, Jonas Manjer, Martin Malmberg, Christer Larsson, Lisa Rydén, Niklas Loman, and Åke Borg

This appendix contains Figure S1 and two SCAN-B protocols, Tumor Sample Processing, and RNA-Sequencing Library Preparation.

(next page)
Figure S1 – Schema for RNA-sequencing library preparation.

SCAN-B directional RNA-seq Library Preparation

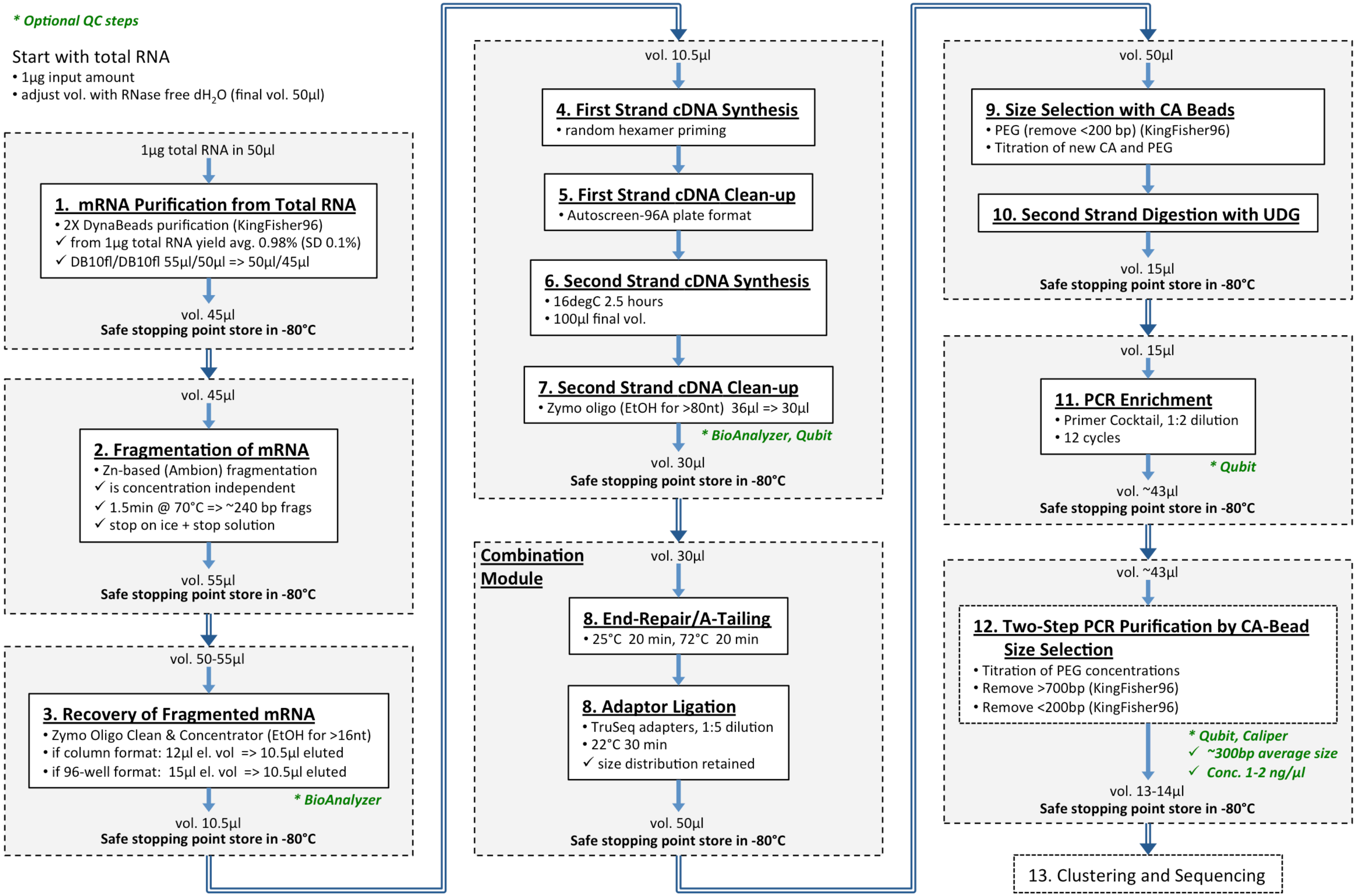

Table of Contents

Section	Page
**Table of Contents**	**1**
**Introduction**	**1**
**1. Tissue Preservation**	**1**
**2. Collection and LIMS Registration**	**2**
**3. Specimen Partitioning**	**3**
**4. Tissue Disruption and Lysis**	**5**
**5. AllPrep RNA/DNA/flow-through isolation using the QIAcube**	**7**
**6. RNA and DNA QC**	**10**
**7. Equipment, Consumables, and Reagents**	**11**

Introduction

This is a custom protocol for the tissue sample treatment of tumor samples for genomic analysis of breast cancer within the SCAN-B Initiative. The procedure is divided into preservation, partitioning, histology, and isolation using the AllPrep method of total RNA for fragments > 200bp, DNA isolation, and possibility for protein isolation and small RNA isolation from the retained flow-through fraction.

Samples: Preserved tumor samples (or fresh/snap frozen specimens), optimal weight: 5-30 mg (min 1 mg, max 50 mg).

1. Tissue Preservation

1.1. Clinical Pathology

1.1.1. Tissues should be transported and manipulated on ice.

1.1.2. Tissue specimens of approximately 3x3x3 mm are collected but a single smaller piece may suffice. For efficient preservation, the widest dimension of a collected sample should not exceed 5 mm.

1.1.3. Place specimen(s) in pre-aliquoted SCAN-B tubes (Corning 2.0 ml cryogenic vials, cat# 430659) containing 1 ml RNAlater (at least 5 parts RNAlater to 1 part specimen).

1.1.4. Use separate tubes for multiple specimens if they are considered separate samples, e.g., a multifocal tumor or a lymph node. Annotate details on barcode stickers and referral form.

1.1.5. Apply barcode sticker and fill in SCAN-B pathology form.

1.1.6. Place tube in 4°C and record date/time. RNAlater should be allowed to penetrate specimen for at least 16 hours prior to processing or freezing.

1.1.7. Ship to Lund Pathology following schedule and using inter-hospital transport.

1.1.8. At Lund Pathology, all SCAN-B specimen tubes are placed in Lund SCAN-B 4°C refrigerator to await pick-up by SCAN-B personnel.

1.1.9. Tested RNA stability after the 16h @ 4°C incubation:

Temperature	Stability
4°C	= up to 4 weeks (we aim to store at 4°C for < 5 days)
RT	= up to 5 days
-80°C	= long term

2. Collection and LIMS Registration

Specimens and referral forms are delivered in cold-pack insulated carrier bags.

2.1. Collection and Registration in LIMS

2.1.1. Match collected specimen tubes with SCAN-B pathology forms. If a pathology form or specimen tube is missing, notify the clinical liaison.

2.1.2. Note time of surgery and check which specimens can be partitioned the same day. Specimens should be stored at 4°C for at least 16 hours before partitioning or freezing.

2.1.3. Log in to: *BASE → Extensions → Reggie → Sample processing wizards → Specimen tube registration.*

2.1.4. *Enter Case Name (step 1 of 3)*. Use the barcode reader to enter Case name (“studie löp nr”) → *Next* to proceed.

2.1.5. *Enter Case information (step 2 of 3)*. Fill in: Number of tubes, Arrival date, Sampling date and time, RNA Later date and time, Laterality, Specimen type, Biopsy type, Other path note; → *Next* to proceed.

2.1.6. *Enter tube information (step 3 of 3)*. Assigned box number in freezer will come up. Fill in delivery comment → *Create* to save.

2.1.7. When the referrals have been registered in BASE, leave them in the "UT" tray. Unregistered referrals may be left in the "IN" tray. *Note: The referrals must always be kept in the secure location.*

2.1.8. Go to the Brady computer and log in to *BASE* → *Extensions* → *Reggie* → *Sample processing wizards* → *Partition registration wizard*. Select specimen tubes that you want to process → *next* → *download label files*. Save downloaded file on desktop as CSV with the date as filename (e.g., 20120221.csv).

2.1.9. Open the software Multiple_Brady_Printer → *Data sources* → *Database* → *Create*. Under *Select a datasource* choose csv and select the file that was downloaded and saved on the desktop → *ok*.

2.1.10. In software Multiple_Brady_Printer, Select *Print direct* under Print to check if the number is in the correct position on the printed labels, preferably as far down as possible. If needed change position on screen or move the label in the printer. When it is ok select → *Print* → *More* → *all records* → *ok*.

2.1.11. Collect printed labels for lab tubes: one for AllPrep, Histology, and the Remainder.

3. Specimen Partitioning

Optimally we process a ~30 mg piece of each specimen for extraction of nucleic acids using the AllPrep method. The tumor pieces are delivered in tubes containing 1 ml of RNAlater. Store them at 4°C for at least 16 hours from operation time to allow full penetration of tissue with the preservative. All received samples must be properly registered in BASE prior to sample processing.

3.1. Before you start

3.1.1. Spray lab benches with Ambion RNAse Away (cat# 10328-011) and wipe them dry.

3.1.2. Put a sterile blanket on the bench; change when needed (if possible every day).

3.1.3. Prepare 2.0 ml Eppendorf Safe-Lock tubes with pre-printed barcoded labels for AllPrep, Histology, and Remainder. Add 500 µl RNAlater to tubes for Histology and Remainder (no RNAlater for AllPrep tubes). *Write last 3 digits of barcode on top of tubes.*

3.1.4. Prepare for tissue dissection by having petri dishes (35 x 10 mm) and single use scalpels (blade no. 10) for each tissue sample to be dissected.

3.2. Partitioning

3.2.1. Log in to the laptop next to the scale (partitioning lab bench), "wake" the scale by pressing any button. Press the green icon (Metler Toledo Balance link software) select → *Configuration → interface → Port, COM3 →ok.*

3.2.2. Log in to *BASE → Extensions → Reggie → Sample processing wizards → Partition registration wizard.* Select specimens to be partitioned and press → *Next*.

3.2.3. Put fresh petri dish on the bench and get the first tissue piece out of its tube and onto the dish. Make note of the appearance of the tumor and enter the *Number of pieces* (NofPieces) in the BASE form.

3.2.4. Cut off desired sections with a scalpel. In priority order: one representative piece for AllPrep (~30 mg), one section immediately adjacent to be used for Histology (5-10 mg), and one for Remainder (if there is any left). The AllPrep piece should be subpartitioned into smaller pieces to enhance tissue lysis. Use single-use scalpels and forceps to handle each sample. *Note: check the box 'Mult' if sampling multiple pieces is required to get material for AllPrep.*

3.2.5. If the delivered specimen is <15 mg, then only take a piece for AllPrep. If the specimen is exactly 15 mg, then take 10 mg to Allprep and 5 mg to Histology.

3.2.6. Dispose of used single-use utilities and discard the scalpels in the yellow sharps box.

3.2.7. Repeat for each tissue sample.

3.2.8. As prompted by BASE, weigh each tube containing the 500 µl RNAlater, tare, and place the appropriate cut section(s) into the labeled tube, and record in BASE by pressing print on the scale.

3.2.9. If Histology piece cannot be sampled, write 0 in the HisWeight field.

3.2.10. Place the tubes with the pieces for AllPrep, Histology, and Remainder in their assigned storage locations in the -80°C freezer (AllPrep and Remainder) or 4°C fridge (Histology).

4. Tissue Disruption and Lysis

Tissue samples are lysed and homogenized with the TissueLyzer (TL) and the RNA/DNA is extracted using the AllPrep method, automated on the QIAcube (all from Qiagen). Flow-though fraction is saved for eventual isolation of small RNAs and proteins.

4.1. Before you start

4.1.1. Log in to *BASE → Extensions → Reggie → Sample processing wizards → DNA/RNA extraction wizards → Lab Tracking Protocol for Allprep isolation*.

4.1.2. Select the 12 samples that are next in line under *Select unprocessed lysate items → Finish → Print*.

4.1.3. Make sure that the samples selected are in the AllPrep storage box in the -80°C freezer and check the "ApWeight" (printed in remark). If any sample is less than 10 mg or more than 50 mg this sample should be treated differently (described later in this protocol).

4.1.4. Chill the TissueLyser (TL) adapter at -20°C for at least 1 hour.

4.1.5. Take out samples from freezer to thaw at room temperature if they are in RNAlater. If fresh frozen tissue is being used, it should at all times be on dry ice.

4.1.6. Retrieve the remaining pre-printed labels for all samples to be processed.

4.2. Prepare lysis buffer

4.2.1. Always use Eppendorf 2.0 ml Safe-Lock tubes for disruption/lysis. Mark two tubes/sample:

- One with the label ending with ".l" (lysate) and the number of the sample on the lid (together with position in Qiacube).
- The other with the last three numbers of the sample on the side and the QIAcube number on the lid (for AllPrep isolation)

4.2.2. From this step work on the ventilated bench. Prepare the "Lysis buffer mix" (800 μl/sample). Mix 790 μl RLT Plus lysis buffer + 8 μl 2-Mercaptoethanol (BME). Make a lysis mastermix for 13 samples if you are processing 12 samples (10,270 μl RLT + 104 μl BME in a 15 ml tube). Be aware that, if any of the samples are over 50 mg, additional buffer will be needed; therefore check this before preparing the mastermix.

4.2.3. Rinse the steel beads (5 mm) to be used (2 per sample) in 1 ml fresh RLT Plus lysis buffer using a 2 ml tube and a clean pair of forceps (maximum 12 beads in one tube).

4.3. Tissue disruption

4.3.1. Quickspin the thawed sample tubes and remove the RNAlater solution (~600 µl). Be sure that all the salt precipitate is dissolved.

4.3.2. Add 2ul Reagent DX-Antifoaming reagent to each sample.

4.3.3. Place 2 steel beads in each sample tube and store the samples in the pre-cooled TL adapter (12 samples/run).

4.3.4. Add 400 µl lyses buffer mix and disrupt the samples in the TissueLyser, disrupt the samples at 50 Hz for 2 x 4 minutes (pause in-between).

4.3.5. After disruption centrifuge the samples briefly and keep at room temperature

4.3.6. For samples 10-50 mg, add an additional 400 µl lysis buffer and mix well

4.3.7. For samples <10 mg, do not add additional lysis buffer.

4.3.8. For samples >50 mg, add 600 µl lysis buffer.

4.3.9. Add up to 800 µl lysed sample to the pre-labeled QIAshredder columns.

4.3.10. Centrifuge at 16000 g for 5 minutes at room temperature. Spin longer if precipitate appears loose in order to pellet it.

4.3.11. Flow-through contains RNA/DNA and protein. Continue with 350 µl of flow-through lysate for RNA/DNA isolation in a new tube, and store the remaining ~390 µl in a new labeled tube for storage (suffix ".l"), taking care to leave behind any precipitate. If the specimen was <10 mg, then there will be no lysate for storage. If the specimen was >50 mg, then repeat loading the same QIAshredder column and split the flow-through such that 350 µl is used immediately for RNA/DNA isolation and the remainder lysate is stored for future use.

4.3.12. Store the homogenized samples for at least 30 minutes at -80°C or on dry ice before continuing with RNA/DNA isolation.

4.3.13. The homogenized samples for storage should be put in the "lys" box in the position indicated by BASE.

4.3.14. Dispose of BME waste appropriately.

5. AllPrep RNA/DNA/flow-through isolation using the QIAcube

This is the protocol for isolation of RNA and DNA from 350 µl homogenized tissue sample in RLT Plus buffer using the AllPrep method. Customized QIAcube protocols were developed for SCAN-B which modified slightly the standard protocols provided by Qiagen. Additional instructions are found in the QIAcube Protocol Sheet and in the Customized Protocol General Information ID 1608 v2. Protocols are available under:

RNA → AllPrep DNA/RNA Mini Kit → Animal tissues and cells →
Part A (DNA Purification): → Custom part A
Part B (protein/smallRNA FT):→ AllPrep Mod 1 part B
Part C (RNA Purification): → AllPrep Mod 1 part C

5.1. Preparation

5.1.1. Thaw the samples processed above and bring them to RT. Continue using the "Lab Tracking Protocol for Allprep isolation" printed out from BASE.

5.1.2. Retrieve the labels previously printed out.

5.1.3. The label ending with ".d" (DNA) and label ending with ".r" (RNA) should go on 1.5 ml safe-lock tubes (cat# , Eppendorf), and mark the lids with the sample ID.

5.1.4. The last label, ending with ".ft" (flow through) should be put on a 1.5 ml protein low-binding safe-lock tube (cat# , Eppendorf). Mark the lid with the number.

5.1.5. Mark (using pen) two Rotor adapter and two 1.5 ml collection tubes (from the AllPrep kit) for each sample, with QIAcube position number. Put them on two separate rotor adapter holders.

5.1.6. Put marked collection tube in position 3 on respective Rotor adapter and fasten lid in position L3 (see picture below or "Protocol sheet").

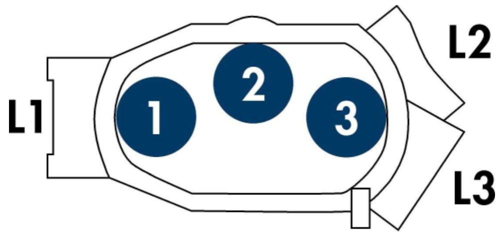

5.1.7. Fill up with Filter-tips (1000 µl) in the QIAcube.

5.1.8. Prepare one 2 ml safe-lock tube (cat# Eppendorf) with RNAse free water (for 12 samples 1336 µl) for the RNA elution step and put the tube in slot A in the QIAcube.

For fewer samples than 12 see volume “Protocol Sheet”. It is important to have the exact volume that is described in the protocol sheet.

5.1.9. Check the “QIAcube-AllPrep-Buffer Exchange List” if any reagents needs to be exchanged. Put the bottles in the Reagent Bottle Rack in correct position (see Protocol Sheet). Fill up with solutions from kit if needed (it should be at least 2/3 in each bottle). Note: mark the bottle with date if a new bottle is opened.

5.1.10. Remove the caps from the reagent bottles and put them in the assigned box. Put the reagent bottle rack in the QIAcube.

5.1.11. Cut the lids off the AllPrep DNA spin columns.

5.1.12. Work on the ventilated bench after running each QIAcube program.

5.2. Part A (DNA isolation)

5.2.1. Place the labeled elution tubes in position 3 and the lidless AllPrep DNA spin columns into position 2 of each rotor adapters. (Position 1 is empty).

5.2.2. Place the rotor adapters into the QIAcube centrifuge.

5.2.3. Quickspin the thawed samples lysates and place them in their correct positions in the QIAcube shaker.

5.2.4. Close the QIAcube and start the program → Custom part A. Run time is approximately 30 minutes for 12 samples. *Note: This is a modified program; importantly an additional 1 minute incubation following addition of wash buffer was added.*

5.2.5. At the end of the program take out the rotor adapters and place into the rotor adapter holder.

5.2.6. Remove the DNA spin column and save the labeled DNA elution tube on ice.

5.2.7. Transfer the eluted DNA to a new labeled 1.5 ml safe-lock DNA tube.

5.2.8. Transfer the flow-through (containing total RNA and protein) from the *used rotor adapter Position 2* (approximately 310 μl) into *Position 2 of a fresh rotor adapter*.

5.3. Part B (collection of flow-through for protein/smallRNA)

5.3.1. Prepare a second set of Rotor adapters.

5.3.2. Place clean RNeasy spin columns in position 1 with the lid fastened in position L1 of the new adapters.

5.3.3. Place RNA elution tubes into position 3 of the new adapters.

5.3.4. Transfer the flow-through (containing total RNA and protein) from the *used rotor adapter Position 2* (~310 µl) from above into *Position 2 of the new rotor adapter*.

5.3.5. Place the rotor adapters into the correct positions in the QIAcube.

5.3.6. Close the QIAcube and start the program → AllPrep Mod 1 part B. Run time is approximately 13 minutes for 12 samples. *Note: This is a modified program to allow for saving of the flow-through which contains the protein and small RNA fractions.*

5.3.7. At the end of the program take out the rotor adapters and place into the rotor adapter holder.

5.3.8. Save the protein/small RNA flow-through (550 µl) from the *space between the adapter positions* and transfer to a new pre-labeled 1.5 ml protein low-binding safe-lock tube and store at -80°C according to position indicated by BASE.

5.3.9. Place the rotor adapters back into the QIAcube maintaining the same centrifuge adapter holder positions as before.

5.4. Part C (complete RNA isolation)

5.4.1. Close the Cube and start the program → AllPrep Mod 1 part C. Run time is approximately 23 minutes for 12 samples.

5.4.2. At the end of the program take out the rotor adapters and place into the rotor adapter holder.

5.4.3. Remove the RNeasy spin columns and save the labeled RNA elution tubes on ice.

5.4.4. Transfer the eluted RNA into a new pre-labeled 1.5 ml safe-lock tube and store on ice.

5.4.5. Take out reagent bottles and put the lids on to prevent ethanol evaporation.

5.4.6. Clean the QIACube with 70% ethanol if needed and then clean out used tips from drawer and clean the inside of the drawer with 70% EtOH and put it back again. Dispose of waste appropriately.

5.4.7. Store all samples at -80°C in labeled boxes and in positions as directed by BASE.

6. RNA and DNA QC

6.1. Nanodrop

6.1.1. Directly after isolation of RNA and DNA, log in to the 8-channel Nanodrop (ND-8000) computer and log in to *BASE → Extensions → Reggie → Sample processing wizards → DNA/RNA extraction wizards → DNA/RNA/Flowthrough registration*.

6.1.2. Select the 12 samples that have been extracted under Select unprocessed lysate items → *Next*; fill in information on Lysis and Qiacube run under Common information from Lysate and Qiacube step → *Next*.

6.1.3. Under *RNA/DNA/Flowthrough details → NanoDrop Sample ID → click Download* → save file on desktop (remove file when finished).

6.1.4. Open the ND-8000 program and upload the file when prompted (Load Sample ID file). The DNA samples are now listed on row 1 and 2 and the RNA samples are listed on 3 and 4. *If any of the samples get an error message or look strange do the re-measurement in the same position. Measurements that are done in other positions will not be uploaded into BASE.*

6.1.5. After measurement of samples save the report as a “txt” file on the server *sky1:\scanb\SCAN-B\Nanodrop_resultat* in folder with appropriate “month-year”. Name the file with “date of extraction + _DNA_RNA”. Print the file.

6.1.6. Go back to *BASE Extensions → Reggie → RNA/DNA/FlowThrough details → NanoDrop values → click ‘Browse’ → Select the results file from ND measurement → open.* The concentrations should now appear for each sample.

6.1.7. Check that the values are the same as on the ND results file. If any of the samples have been re-measured BASE will automatically take the measurement with the highest value, *if this is not correct change the value in BASE*.

6.1.8. Check that the lysate volumes are correct. If samples had a weight < 10 mg the Lysate *Total* is only 350 µl and if a sample had a weight > 50 mg the Lysate *Total* is 900 µl. For normal samples the lysate is 700 µl.

6.1.9. If anything needs to be changed or a comment is to be added *press → Edit… → change what’s needed → ok → Finish.*

6.2. Preparation of RNA aliquots for Caliper run

6.2.1. In *BASE* → *Reggie* → *Sample processing wizards* → *RNA quality control wizards* → *Create aliquots on Bioanalyzer/Caliper plates* → Select the RNA extracts to be aliquoted → *Next.*

6.2.2. Positions of samples on Caliper plate and concentrations appear and if the RNA sample concentration is <35 ng/µl the HS (High Sensitivity assay on Caliper) box is ticked.

6.2.3. Write down the positions of the samples on the Caliper plate in the comment box on the "Lab Tracking Protocol for Allprep isolation" for current run. Also note if any of the samples are to be run with the HS assay; press → *Finish*.

6.2.4. Prepare 12-tube strips for RNA quality check on Caliper, write the 3 last numbers of the sample on each tube on the strip. For HS, aliquot 2 µl per sample to the strip. For Std take 2 µl up to 200 ng/µl, and if at a higher concentration then dilute to <200 ng/µl and aliquot 2 µl to the Caliper strip.

6.2.5. Put strip caps on and label them with plate position and the ends with column number and put them in the correct position in the specific Caliper plate in the -80°C freezer.

6.2.6. When the plate is complete it will be run on Caliper. The results are stored directly in BASE. For Caliper instructions see protocol "Caliper LabChip GX HT RNA Assay user instructions".

6.2.7. Store all samples at -80°C in correct positions in labeled boxes as indicated by BASE.

6.2.8. Put the completed "Lab Tracking Protocol for Allprep isolation" with the print out of the NanoDrop results in folder named "SCAN-B QC".

7. Equipment, Consumables, and Reagents

Equipment:

- QIAcube (cat# 9001293, Qiagen)
- TissueLyser LT (cat# 85600, Qiagen)
- Caliper LabChip GX (cat# 122000, PerkinElmer)

Consumables and Reagents:

- Cyogenic vial 2 ml (cat# 430659, Corning)
- Brady label stickers (cat# BPT-628-461, Brady)
- Petri dish 35 x 10 mm (cat# 353001, Falcon)
- Single-use no. 10 scalpels (cat# REF0501, Swann-Morton)
- AllPrep DNA/RNA Mini Kit (cat# 80204, Qiagen)
- Stainless Steel Beads 5 mm (cat# 69989, Qiagen)
- TissueLyser 2x24 adapter set (cat# 69982, Qiagen)
- Reagent DX (cat# 19088, Qiagen)
- Buffer RLT Plus (cat# 1053393, Qiagen)
- RNAlater 500 ml (cat# AM7021, Ambion)
- Safe-lock Tubes 2.0 ml (cat# 0030 123.344, Eppendorf)
- Safe-lock Tubes 1.5 ml (cat# 0030 123.328, Eppendorf)
- Protein low-bind Safe-lock Tubes 1.5 ml (cat# 0030 097.221, Eppendorf)
- 2-Mercaptoethanol >98% (cat# M3148-100ml, Sigma)
- QIAshredder columns (250 pcs) (cat# 79656, Qiagen)
- QIAcube Filter Tips 1000 µl (cat# 990352, Qiagen)
- QIAcube Rotor Adapters (240 pcs) (cat# 990394, Qiagen)
- QIAcube Reagent Bottle (30 ml) (cat# 990393, Qiagen)
- 12-tube strips (200 µl) (cat# 732-0553, VWR)

Table of Contents

Section **Page**

Introduction **1**
1. mRNA Purification from Total RNA **2**
2. Fragmentation of mRNA **5**
3. Recovery of Fragmented mRNA **5**
4. First Strand cDNA Synthesis **7**
5. First Strand cDNA Clean-up **8**
6. Second Strand cDNA Synthesis **8**
7. Second Strand cDNA Clean-up **9**
8. End-Repair/A-Tailing and Adaptor Ligation **11**
9. Size Selection with CA Beads **12**
10. Second Strand Digestion with UDG **14**
11. PCR Enrichment **14**
12. Two-Step PCR Purification by CA-Bead Size Selection **15**
13. Clustering and Sequencing **18**
14. Equipment, Consumables, and Reagents **18**

Introduction

This is the SCAN-B RNA-seq protocol for preparation of Illumina-compatible sequencing libraries from total RNA in a high-throughput format with an integrated workflow together with the BioArray Software Environment (BASE) laboratory information management and analysis web-based platform. The library preparation method is an adaptation of the dUTP strand-specific method as described by Parkhomchuk et al. (*Nucleic Acids Res* 2009) and modified by Lohan and colleagues (Nalpas et al., *BMC Genomics* 2013). Size selection using polyethylene glycol and carboxylic acid (CA)-beads is adapted from described methods by Borgstrom et al. (*PLOS One* 2011).

1. mRNA Purification from Total RNA

This is a modification of the Invitrogen protocol "Dynabeads mRNA DIRECT Kit (rev008)" (cat# 61012) for automatization on the ThermoScientific KingFisher Flex Magnetic Particle Processor (KF-Flex). Input material is 1.1 µg (1-2 µg can readily be used) total RNA diluted in 50 µl nuclease-free water. Two rounds of mRNA purification are performed to reduce ribosomal RNA. For breast cancer samples, the expected yield of mRNA after the 2nd round is approximately 1-2% of total RNA. Relative humidity in the KF-Flex should be stabilized by placing open plates with water inside prior to starting.

1.1. Diluting total RNA samples

1.1.1. Use BASE to create the worksheet for the next batch of total RNA samples to be purified: *BASE → Extensions → Reggie → Library preparation wizards → Lab protocols for mRNA and cDNA preparation*. Select mRNA bioplate and input concentration of the Stratagene Universal Human Reference RNA (cat# 740000, Agilent) being used. Print out List layout and Plate layout.

1.1.2. Dilute all samples in a 96-well 4titude PCR plate, 1.1 µg total RNA in 50 µl nuclease free water.

Note: this is an optional stopping-point: plate can be sealed and stored at -80℃.

1.1.3. Vortex plate and spin it down before incubation.

1.1.4. Incubate the samples at 65°C for 5 min in a thermocycler (e.g. Eppendorf vapo.protect).

1.1.5. Place denatured diluted total RNA samples on ice.

1.2. Preparation of Binding Plates

1.2.1. In advance, contents of Ambion mRNA Purification Kit should be brought to room temperature (allow at least 20 min).

1.2.2. Vortex the Dynabeads and transfer 100 µl Dynabeads per well into the Binding Plate1 (KingFisher 96 plate 200 µl, cat# 97002540).

Note: 100 µl/well Dynabeads is prepared to cover both round #1 and round #2.

1.2.3. Remove suspension buffer by applying plate to 96-position magnetic stand and wait until the solution is completely clear (1-2 min); then discard the supernatant by pipetting.

1.2.4. Wash beads: add 100 µl Binding Buffer and mix by pipetting.

1.2.5. Remove Binding Buffer by applying plate to 96-position magnetic stand, wait until clear (1-2 min), and discard the supernatant by pipetting.

1.2.6. Add 100 µl Binding Buffer and mix by pipetting.

1.2.7. Per well, transfer 50 µl of the resuspended Dynabeads to Binding Plate2 for later use in round #2 (see section below).

1.2.8. Add 50 µl of the diluted total RNA samples to each well in Binding Plate1 and mix by pipetting (total volume 100 µl).

1.3. Preparing KF-Flex for round #1 of mRNA purification

1.3.1. Label and fill the KF 96 plates as follows:

1	- Tip Plate:	Put 1 new KingFisher 96 tip comb on to the TIP Plate
2	- Elution Plate:	55 µl nuclease free water
3	- Wash Plate 2:	60 µl Washing Buffer B
4	- Wash Plate 1:	60 µl Washing Buffer B
5	- Binding Plate:	50 µl prepared Dynabeads + 50 µl prepared Samples (as described above)

1.4. Run KF-Flex, purification round #1

1.4.1. Make sure that the A1 positions on the plates are in the same corner as the A1 positions of the KF-Flex disc.

1.4.2. Start program mRNA_DB10fl, follow on-screen instructions.

1.4.3. After completed run, remove elution plate immediately and place on 96-position magnetic stand and wait until the solution is completely clear (1-2 min).

1.4.4. Transfer 50 µl of the samples (all) into a new PCR plate, seal the plate, and store it on ice until round #2.

Note: when proceeding directly to round #2 samples are already denatured from the elution step, otherwise a separate denaturation should be added by incubation at 65℃ for 5 min in a thermocycler.

1.5. Preparing KF-Flex for round #2

Note: Dynabeads for Binding Plate2 is prepared together with Binding Plate for round #1 (above). Prepare for round #2 while round #1 is running in the KF-Flex.

1.5.1. Label and fill the KF 96 plates as follows (note lower volume in Elution Plate compared to round #1):

1	- Tip Plate:	Put 1 new KingFisher 96 tip comb on the TIP Plate
2	- Elution Plate:	50 µl nuclease free water
3	- Wash Plate 2:	60 µl Washing Buffer B
4	- Wash Plate 1:	60 µl Washing Buffer B
5	- Binding Plate2:	50 µl prepared Dynabeads + 50 µl Sample from round #1

1.6. Run KF-Flex 2nd Round

1.6.1. Make sure that the A1 positions on the plates are in the same corner as the A1 positions of the KF-Flex disc.

1.6.2. Start program mRNA_DB10fl, follow on-screen instructions.

1.6.3. After completed run, remove elution plate immediately and place on 96-position magnetic stand and wait until the solution is completely clear (1-2 min).

1.6.4. Transfer 45 µl of the samples (all) into a new PCR plate, seal the plate, and store it on ice.

Safe stopping point – may store at -80°C

1.7. Comments

After two rounds of mRNA purification using Dynabeads, mRNA yield will be between 1-5% of the total RNA.

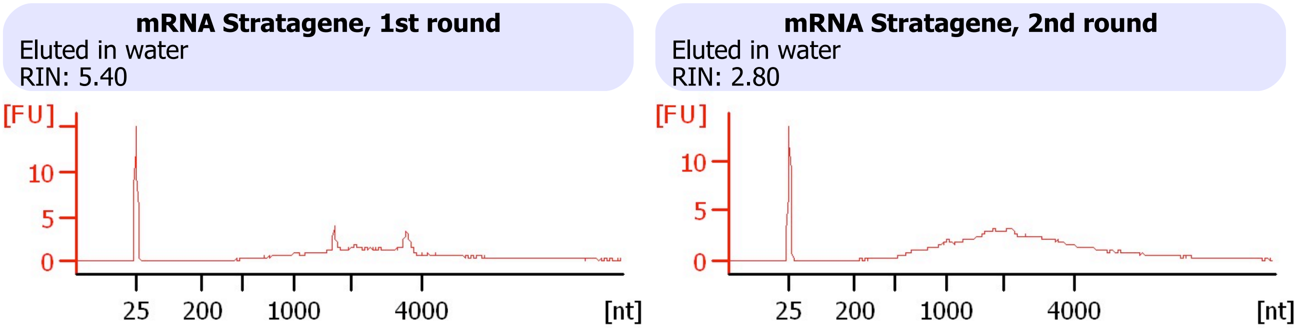

Figure 1. Example BioAnalyzer analysis of mRNA purification after 1 round and 2 rounds.

2. Fragmentation of mRNA

Purified mRNA is fragmented to ~240 bp fragments using the Ambion buffered zinc fragmentation reagents (cat# AM8740). In our hands, incubation for 1.5 minutes yields optimally-sized fragments. Fragmentation is robust to variation in input-RNA concentration and to delay in addition of Stop Buffer as long as samples are put on ice immediately after incubation.

2.1. Fragmentation of mRNA

2.1.1. Assemble the following reaction in PCR tube/strip/plate format:

- mRNA 45 µl
- 10X Fragmentation Reagent 5 µl

2.1.2. Incubate at 70°C for 1.5 minutes in a thermocycler.

2.1.3. Place the tube/plate on ice.

2.1.4. Add 5 µl of Stop Buffer.

Safe stopping point – may store at -80°C

3. Recovery of Fragmented mRNA

The Zymo Oligo Clean & Concentrator with high EtOH (~70%) is used to clean and concentrate fragmented mRNA larger than 16 nucleotides. This can be performed in either column (cat# D4061, Zymo Research) or 96-well plate (cat# D4063) format.

3.1. Recovery of fragmented mRNA

3.1.1. Add 100 µl Oligo Binding Buffer to a new deep well collection plate (from Zymo kit).

3.1.2. Transfer fragmented mRNA (~55 µl) to the collection plate and mix by pipetting.

3.1.3. Add 400 µl **absolute ethanol** to the collection plate with Oligo Binding Buffer and fragmented mRNA, mix briefly by pipetting and transfer mixture to the corresponding well of a Zymo-Spin I-96 Plate.

3.1.4. Centrifuge at 5000 g for 5 minutes. Discard the flow-through.

3.1.5. Add 800 µl Wash Buffer to all the wells on the plate.

3.1.6. Centrifuge the plate at 5000 g for 5 minutes and discard the flow-through.

3.1.7. To completely dry the plate, centrifuge at 5000 g for 7 minutes and discard any flow-through.

3.1.8. Transfer the plate onto an Elution Plate (in this case we use a PCR plate). Add 15 µl water directly to the column matrix (*make sure that the entire volume is on the matrix*) and let the plate stand for 1 minute. Centrifuge at 5 000 g for 7 minutes to elute the mRNA.

3.1.9. Transfer 10.5 µl of the eluate to a new PCR plate.
Note: transfer to a new PCR plate is not required if an appropriate stand/rack is used to support (prevent damage) the PCR plate during centrifugation; however, verify that volumes are even across wells.

3.2. Quality Control (optional)

Quality control of fragmented mRNA can be performed by RNA Pico Chip on the Bioanalyzer. Since an amount of the sample is lost when running quality control, samples must be pre-selected from the start and compensated by inputting an extra amount of total-RNA in the mRNA purification (add an extra 11% of total-RNA).

3.2.1. Aliquot 1.2 µl for selected samples to perform quality control on a RNA Pico Chip on the Bioanalyzer. Add back 1.2 µl water to these samples to compensate the total volume.

3.2.2. Use BASE to register mRNA plate and upload Bioanalyzer PDF file: *BASE → Extensions → Reggie → Library preparation wizards → mRNA registration and quality control results.*

Safe stopping point – may store at -80°C

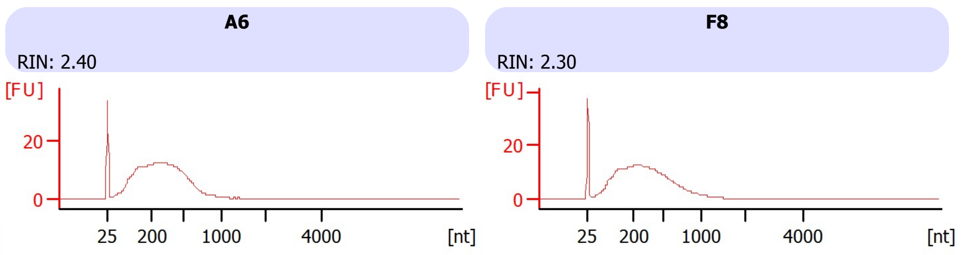

Figure 2. Example of typical QC results for fragmented mRNA from well A6 (left) and F8 (right).

4. First Strand cDNA Synthesis

First strand cDNA is synthesized using random hexamer priming, dNTP mix, and SuperScript II Reverse Transcriptase reagents (cat# 18064-014, Life Technologies).

4.1. Priming

4.1.1. Assemble the following reaction in a PCR tube/PCR plate

- Fragmented mRNA 10.5 µl
- Random hexamers (3 µg/µl) 1 µl

4.1.2. Mix well with a pipette.

4.1.3. Incubate 65°C for 5 minutes in a thermocycler.

4.1.4. Place tube/plate on ice.

4.2. cDNA synthesis

4.2.1. Mix the following 1st Strand Synthesis Mix, per sample (use SCAN-B calculator):

- 5X First Strand buffer 4 µl
- 0.1 M DTT 2 µl
- 10 mM dNTP mix 1 µl
- RNaseOUT (40 U/µl) 0.5 µl

4.2.2. Add 7.5 µl of 1st Strand Synthesis Mix to the mRNA+hexamers sample, mix well (total volume 19 µl).

4.2.3. Place in thermocycler, run the following program:

- 25°C 2 min
- **PAUSE**, add 1 µl SuperScript II, take care to mix well (mix w larger volume)
- Resume program, 25°C 10 min
- 42°C 50 min
- 70°C 15 min
- 4°C Hold

4.2.4. After completed program store samples on ice and proceed directly to clean-up.

5. First Strand cDNA Clean-up

First strand clean-up is performed using the Illustra AutoScreen-96A plate (cat# 25-9005-98, GE Healthcare) to remove unincorporated dNTPs. **Note:** Set out AutoScreen-96A plates ahead of time (~2 hr) as they must be used at room temperature or else performance will be erratic.

5.1. Prepare AutoScreen-96A Well Plate

5.1.1. Remove the AutoScreen-96A from the foil storage pouch.

5.1.2. Remove both the top and bottom adhesive seals and place it directly on a collection plate (U-bottom plate).

5.1.3. Centrifuge for 5 min at 910 g.

5.1.4. Add, drop wise, 150 µl of distilled water.

5.1.5. Centrifuge for 5 min at 910 g.

5.1.6. Discard collection plate and replace it with a fresh PCR plate (4titude PCR plate).

5.1.7. Slowly apply 20 µl of first strand cDNA to the center of the column resin bed in the AutoScreen-96A plate.

5.1.8. Centrifuge the samples for 5 min at 910 g.

5.1.9. Take note of eluted volume for each well. If needed add water to final volume of 16ul.

5.1.10. Store first strand collection plate on ice and proceed immediately to 2nd strand synthesis.

6. Second Strand cDNA Synthesis

Second strand synthesis incorporates dUTP instead of dTTP. To make a 400 µl mix of 10 nM of each nucleotide substituting dUTP for dTTP, take 40 µl of each 100 nM stock of dATP + dGTP + dCTP + dUTP, plus 240 µl H_2O. Prepare nucleotide mix in advance.
Note: for a full 96-well plate a total of 306 µl is typically needed.

6.1. Second strand synthesis

Note: the recipe for 2nd Strand Synthesis Mix includes water for a final reaction volume of 100 µl. However, if elution volumes from first cDNA clean-up are variable, the volume of water in the 2nd Strand Synthesis Mix may be reduced and the final reaction volume adjusted with water after the 1st strand cDNA reaction sample has been added.

6.1.1. Mix the following 2nd Strand Synthesis Mix, per sample (use SCAN-B calculator):

- 5X First Strand buffer 1.3 µl
- 5X Second Strand buffer 20 µl
- 10 mM dUTP+dATP+dGTP+dCTP mix 3 µl
- 0.1 M DTT 1 µl
- DNA Pol I (10 U/µl) 5 µl
- RNaseH (10 U/µl) 0.2 µl
- H_2O 53.5 µl

6.1.2. Chill 2nd Strand Master Mix on ice for at least 5 minutes before aliquoting.

6.1.3. Add 84 µl of 2nd Strand Master Mix to each 1st strand cDNA reaction and mix (final total volume 100 µl).

6.1.4. Incubate at 16°C in a thermocycler for 2.5 hours (no heated lid).

6.1.5. Proceed to second strand cDNA clean-up.

7. Second Strand cDNA Clean-up

Second strand cDNA clean-up is performed using the Zymo Research Oligo Clean & Concentrator and low EtOH (~57%) to concentrate and clean oligonucleotides >80 bp. This can be performed in either column (cat# D4061, Zymo Research) or 96-well plate (cat# D4063) format.

7.1. Second strand cDNA clean-up

7.1.1. Add 200 µl Oligo Binding Buffer to a new collection plate.

7.1.2. Transfer 100 µl sample to the collection plate containing Oligo Binding Buffer.

7.1.3. Add 400 µl **absolute EtOH** to the collection plate with sample and Oligo Binding Buffer, mix briefly by pipetting and transfer mixture to the corresponding well of a Zymo-Spin I-96 Plate.

7.1.4. Centrifuge at 5000 g for 5 minutes. Discard the flow-through.

7.1.5. Add 800 µl Wash Buffer to all the wells on the plate.

7.1.6. Centrifuge the plate at 5000 g for 5 minutes and discard the flow-through.

7.1.7. To completely dry the plate, centrifuge at 5000 g for 7 minutes and discard any flow-through.

7.1.8. Transfer the plate onto an Elution Plate (in this case we use a PCR plate). Add 34 µl water directly to the column matrix (*make sure that the entire volume is on the matrix*) and let the plate stand for 1 minute. Centrifuge at 5 000 g for 7 minutes to elute the cDNA.

7.1.9. Verify that volumes are even across wells, or transfer 30 µl to a new PCR plate.

7.1.10. Use BASE to register cDNA plate: *BASE → Extensions → Reggie → Library preparation wizards → cDNA registration.*

7.2. Quality Control (optional)

7.2.1. For selected standard samples (e.g. Stratagene Reference RNA) measure concentration using Qubit (will only consume 1 µl of sample, replace used volume with water). Concentrations can be tracked over time across multiple plates for quality assurance.

7.2.2. For selected standard samples (e.g. Stratagene Reference RNA) run BioAnalyzer (will only consume 1 µl of sample). Results can be tracked over time across multiple plates for quality assurance.

Safe stopping point – may store at -80°C

8. End-Repair/A-Tailing and Adaptor Ligation

As a combination module, end-repair, A-tailing, and adapter ligation are performed in an additive reaction. Illumina TruSeq barcoded adapters kits A and B (FC-121-2001 and FC-121-2002) are used, with the adapters first diluted 1:5. Use BASE to assign barcoded adapters samples: *BASE → Extensions → Reggie → Library preparation wizards → Assign barcodes to cDNA plate,* select cDNA bioplate and appropriate preconfigured barcode layout. Use BASE to create worksheets for barcoded adapters: *BASE → Extensions → Reggie → Library preparation wizards → Lab protocols and files for library preparation,* select cDNA bioplate and Print out List layout and Plate layout.

8.1. End-Repair/A-Tailing

8.1.1. Make an end-repair/A-tailing master mix, per sample (use SCAN-B calculator):

- 10X T4 Ligase Buffer 4 µl
- 10 mM dNTP mix 2 µl
- ATP (10 mM) 1 µl
- T4 DNA pol (5U/ul) 1 µl
- T4 PNK (10U/ul) 1 µl
- Taq DNA pol 1 µl

8.1.2. Take 10 µl of mastermix to 30 µl cDNA, mix well (final volume 40 µl).

8.1.3. Place in thermocycler, run the following program:

- 25°C 20 min
- 72°C 20 min
- 12°C Hold

8.2. Adaptor Ligation

8.2.1. The adaptors are first diluted 1:5 with water and we have 24 different adaptors available from Illumina TruSeq DNA LT Sample Prep Kit A and B.

8.2.2. Make an adapter ligation master mix, per sample (use SCAN-B calculator):

- T4 DNA Ligase (5 U/µl) 3 µl
- 10X T4 DNA Ligase buffer 1 µl
- H_2O 5 µl

8.2.3. Add 1 µl of diluted adaptors to each 40 µl sample

8.2.4. Add 9 µl of the adaptor ligation mix, mix well, final volume of 50 µl.

8.2.5. Place in thermocycler, run the following program:

- 22°C 30 min
- 4°C Hold

8.2.6. Proceed to size selection.

Safe stopping point – may store at -80°C

9. Size Selection with CA Beads

Size selection is performed using polyethylene glycol 8000 (PEG) at appropriate (titrated) final concentration and carboxylic acid (CA)-beads to remove fragments <200 bp. We prepare a PEG stock solution (40%) that is sterile-filtered (0.22 µm filter) and aliquoted in 15 ml tubes: we aliquot 13 ml in each tube to cover use for both size-selection and 2-step PCR purification. Aliquot tubes are labeled with preparation date, PEG lot# and stored at 4°C protected from light. Before first use, each new PEG stock solution must be titrated to evaluate size-selection properties to determine appropriate final working concentration, as this can vary between stock solutions.

9.1. Prepare Samples

9.1.1. Add appropriate volume of H_2O to the wells (1-2 µl) to bring up to 50 µl.

9.1.2. Store the samples on ice until the KF96-Binding plate has been prepared.

9.2. Prepare PEG mastermix and 80% EtOH

9.2.1. Vortex a new PEG stock-solution tube (15 ml tube) vigorously. Let sit for several minutes (in dark). The PEG stock-solution is going to be used for both size selection and for 2-step PCR purification step so calculate required volume and make sure that enough volume in available.

9.2.2. Make fresh 40 ml 80% EtOH, by taking 32 ml absolute EtOH + 8 ml H_2O.

9.3. Prepare PEG mastermix

9.3.1. Prepare the mastermix, **MM**, using JVC-1-step-CA-MM-calculator-v2.0.xlsx to calculate the master mix.

Note: the mastermix is prepared using PEG stock-solution, 5M NaCl stock-solution, and water to permit preparation of a binding reaction (final volume 100 µl) by combining sample (50 µl) and mastermix (50 µl) to achieve an appropriate final PEG concentration at 0.9M NaCl. The final PEG concentration in the binding reaction will vary with every new batch of PEG stock-solution and is typically in the range of 7-10% PEG.

9.4. Preparing KF-Flex for size selection run

9.4.1. Allow sufficient time to allow the beads to equilibrate to RT. Shake the magnetic beads bottle for at least 30 min at RT (Heidolph Vibramax 100 Shaker, 300 rpm).

9.4.2. Label and fill the KF 96 plates as follows:

1	- Tip Plate:	Put 1 new King Fisher 96 tip comb on to the TIP Plate
2	- Elution Plate:	15 µl EB
3	- Wash plate:	180 µl fresh 80% EtOH
4	- Binding Plate:	50 µl **MM** + 50 µl sample, mix by pipetting (thorough mixing is crucial)
5	- CA bead plate	40 µl CA beads + 160 µl EB

9.5. Run KF-Flex

9.5.1. Make sure that the A1 positions on the plates are in the same corner as the A1 positions of the KF-Flex disc.

9.5.2. Start program CA_HP6fl (it will take around 23 minutes), follow on-screen instructions.

9.5.3. After completed run, remove elution plate immediately and place on 96-position magnetic stand and wait until the solution is completely clear (1-2 min).

9.5.4. Transfer 13 µl of sample from the elution plate into a UDG plate (prepared in advance) containing UDG master mix, mix well, and store it on ice.

9.5.5. The Binding Plate can be sealed and stored at 4°C and used for further analyses.

Prepare Second Strand Digestion with UDG while the KF-Flex is running.

10. Second Strand Digestion with UDG

The second strand with incorporated dUTP is specifically digested using uracil-DNA glycosylase (New England Bio Labs, cat# M02805).

10.1. Digestion with Uracil-DNA Glycosylase

10.1.1. Make an UDG master mix, per sample (use SCAN-B calculator):

- 10X UDG Buffer 1.5 µl
- UDG (5 U/µl) 0.1 µl
- H_2O 0.4 µl

10.1.2. Take 2 µl of the master mix to a new PCR plate and transfer 13 µl of DNA from CA purification (final volume 15 µl) and store on ice. Mix well.

10.1.3. Place in thermocycler, run the following PCR program:

- 37°C 15 min
- 94°C 10 min
- 4°C Hold

Safe stopping point – may store at -80°C

11. PCR Enrichment

Single-stranded cDNA is amplified by PCR.

11.1. PCR enrichment mastermix

11.1.1. Make a PCR enrichment mastermix, per sample (use SCAN-B calculator):

- Illumina Primer Cocktail (1:2 dilution) 2.625 µl
- 10 mM dNTP mix 0.9 µl
- Phusion Mix (NEB) 22.5 µl
- H_2O 4.875 µl

11.1.2. Add 30.9 µl of PCR mastermix to 15 µl UDG digested cDNA, final volume of 45.9 µl.

11.1.3. Place in thermocycler, run the following PCR program:

- 98°C 3 min
- 12 cycles of: 98°C 30 sec, 60°C 30 sec, 72°C 30 sec
- 72°C 10 min
- 4°C Hold

11.1.4. After PCR place sample tube/plate on ice.

11.1.5. Proceed to two-step purification with CA size selection.

11.2. Quality Control (optional)

11.2.1. For selected standard samples (e.g. Stratagene Reference RNA) measure concentration using Qubit (will only consume 1 µl of sample). Concentrations can be tracked over time across multiple plates for quality assurance.

Safe stopping point – may store at -20°C

12. Two-Step PCR Purification by CA-Bead Size Selection

The PCR product undergoes two cycles of size selection using CA-beads and varying concentrations of PEG, first to exclude DNA fragments >700 bp and then to exclude fragments <200 bp. Due to variations in ambient humidity and temperature and between batches of PEG preparations, this two-step PCR purification should always be done first with QC libraries (e.g. Stratagene Reference RNA, cat# 740000, Agilent) with different PEG concentrations to determine the optimal solution to proceed with for the entire plate of sample libraries on the same date.

12.1. Sample preparation

12.1.1. Make sure that the sample volume (PCR product) is at least 43 µl (expected volume ~45 µl). Top off wells with water if necessary.

12.2. PEG Mastermix-1 (MM-1) and Mastermix-2 (MM-2)

12.2.1. Note: The volumes will vary with every new batch of PEG solution. Prepare the mastermixes, **MM-1** and **MM-2** using JVC-2-step-CA-MM-calculator-v5.0.xlsx. *Note: MM1 is prepared to permit set-up of the 1st binding reaction (final volume 80 µl) by combining sample (43 µl) and MM1 (37 µl) to achieve an appropriate final PEG concentration at 0.9M NaCl. Conversely, MM2 is prepared to permit set-up of the 2nd binding reaction (final volume 160 µl) by combining sample (70 µl from 1st binding reaction) and MM2 (90 µl) to bring final PEG concentration to an appropriate level at 0.9M NaCl. Note: final PEG concentrations in the binding reactions will vary with every new batch of PEG stock-solution and is typically in the*

range of 7-9% for the 1st binding reaction and in the range of 8-12% in the 2nd binding reaction.

12.3. Preparation of KF-Flex 2-step run, step 1

12.3.1. Place the beads at RT for at least 2 hours. Shake the bottle with the magnetic beads for at least 30 min at RT (Heidolph Vibramax 100 Shaker, 300 rpm)

12.3.2. Label and fill the KF 96 plates as follows:

1	- Tip Plate:	Put 1 new King Fisher 96 tip comb on to the TIP Plate
2	- Binding Plate:	37 µl **MM-1** + 43 µl sample, mix by pipetting (thorough mixing is crucial)
3	- CA Bead Plate:	40 µl CA beads + 160 µl EB, mix by pipetting

12.4. Run KF-Flex 2-step, first step

12.4.1. Make sure that the A1 positions on the plates are in the same corner as the A1 positions of the KF-Flex disc.

12.4.2. Start program CA2ST_C_fl, follow on-screen instructions.

12.4.3. After completed run, remove plates immediately and prepare for KF-Flex step 2 run.

12.4.4. Store the **Binding plate** that will be used in the second step run.

12.5. Preparation of KF-Flex 2-step run, step 2

12.5.1. Label and fill the KF 96 plates as follows:

1	- Tip Plate:	Put 1 new King Fisher 96 tip comb on to the TIP Plate
2	- Elution Plate:	15 µl EB
3	- Wash Plate:	180 µl fresh 80% EtOH
4	- Binding Plate:	Binding plate from step 1 run + 90 µl **MM-2**, mix by pipetting (thorough mixing is crucial)
5	- CA Bead Plate:	60 µl CA beads + 140 µl EB, mix by pipetting

12.6. Run KF-Flex 2-step, second step

12.6.1. Make sure that the A1 positions on the plates are in the same corner as the A1 positions of the KF-Flex disc.

12.6.2. Start program CA2ST_B_fl, follow on-screen instructions.

12.6.3. After completed run, remove elution plate immediately and place on 96-position

magnetic stand and wait until the solution is completely clear (1-2 min).

12.6.4. Transfer 13-14 µl (all) of the purified library samples in a new low-binding deep-well Eppendorf PCR plate (cat# 0030503104), seal, and store on ice.

12.6.5. The Binding Plate can be sealed and stored at 4°C and used for further analyzes.

12.6.6. Run the Bioanalyzer DNA High Sensitivity Chip (cat# 5067-4626) on tester Stratagene Reference RNA libraries to verify which PEG concentration should be used for the entire plate.

12.7. Quality Control (optional)

12.7.1. Qubit: 1 µl to Qubit 2.0 Fluorometer (cat# Q32866, Life Technologies). Measure with Qubit prior to aliquoting to Caliper. If the samples have a concentration <500 ng/ml, SpeedVac (Savant) them down to 8 µl, measure once again on Qubit, then aliquot to Caliper.

12.7.2. Caliper: Prefill a blue frame 4titude PCR plate (cat# 4TI-0960/B) with 20 µl nuclease free water; add 2 µl of the samples. Run Caliper according to protocol: Caliper HT DNA High Sensitivity Labchip GX Assay (cat# CLS760672, PerkinElmer) user instruction.

Safe stopping point – may store at -20°C

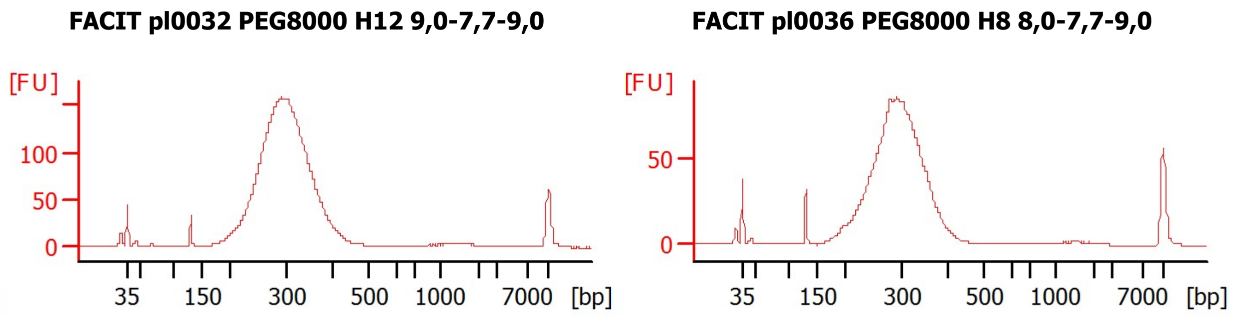

Figure 3. Example of typical QC results for Stratagene Reference RNA libraries from well H12 (left) and H8 (right); size-selection at 9% and 8% (left and right, respectively) and 2-step purification at 7.7% followed by 9%.

13. Clustering and Sequencing

We use BASE to track libraries and pooled libraries and to create protocols for pooling. Concentration and fragment size for libraries are registered in BASE and used to dynamically create pooling recipes. Libraries are diluted to 2nM and pooled according to pre-configured layouts; typically 21 libraries are pooled together.

- Use BASE to register quality control for Stratagene Reference RNA test samples: *BASE → Extensions → Reggie → Library preparation wizards → Register quality control results*.
- Use BASE to register results from Qubit and Caliper results for the whole plate: *BASE → Extensions → Reggie → Library preparation wizards → Library registration*.
- Use BASE to create pooling schema for libraries according to pre-configured layouts: *BASE → Extensions → Reggie → Pooling wizards → Create pooled libraries*.
- Use BASE to generate and download pooling protocols: *BASE → Extensions → Reggie → Pooling wizards → Lab protocols for pooling*.
- Register library pools: *BASE → Extensions → Reggie → Pooling wizards → Register pooled libraries*.

We use *Clustering* and *Sequencing* wizards in BASE to track pools when clustering flow-cells and to track flow-cells through sequencing. Typically each pool is clustered on a total of 4 lanes across 2 separate flow-cells (2 lanes per flow-cell). Pools are diluted to 12 pM according to Illumina's standard procedure and spiked with 0.5% PhiX control. We typically achieve a cluster density between 800-900 K/mm^2 for a total number of PF clusters per lane between 180-200 M (HiSeq 2000).

14. Equipment, Consumables, and Reagents

Equipment:

- KingFisher Flex Magnetic Particle Processor (KF-Flex; ThermoScientific)
- PCR themocycler (Eppendorf vapo.protect)
- 96-positition magnetic stand, Dynal MPC-96S

- Sigma centrifuge 4K1S
- BioAnalyzer
- NanoDrop
- Qubit
- Caliper LabChip GX

Consumables:

96-well 4titude PCR plate	Saveen Werner	4ti-0740
KingFisher 96 plate 200 µl (Elution-, Wash-, and Binding Plate) ???	VWR	FINN97002540
KingFisher 96 tip comb	VWR	733-3015
Low-binding deep-well Eppendorf PCR plate	VWR	0030503.104

Reagents:

**mRNA purification**		
Dynabeads mRNA Purification Kit	Life Technologies	cat# 61006
Column-based Oligo Clean & Concentrator	Zymo Research	cat# D4061
RNA Fragmentation Reagents	Ambion	cat# AM8740
Oligo Clean & Concentrator (single columns)	Zymo Research	cat# D4061
ZR-96 Oligo Clean & Concentrator	Zymo Research	cat# D4063
**First strand cDNA Synthesis and Clean-up**		
Random hexamer	Invitrogen	Custom Oligo NNNNNN, 10U
SuperScript II Reverse Transcriptase	Life Technologies	cat# 18064-014
Superscript II RT 1000U	Invitrogen	cat# 18064014
5X First strand buffer and 0.1 M DDT provided with SuperScript II Reverse Transcriptase		
RNase OUT 40U/ul	Invitrogen	cat# P/N 100000840
10 mM dNTP	Thermo Scientific	cat# R0192
Illustra AutoScreen-96A plate	GE Healthcare	cat# 25-9005-98
**Second Strand cDNA Synthesis**		
5X First strand buffer and 0.1 M DDT provided with SuperScript II Reverse Transcriptase		
5X Second-Strand Buffer	Life Technologies (Invitrogen)	cat# 10812-014
dATP 10mM	Thermo Scientific	cat# R0141
dUTP 10mM	Thermo Scientific	cat# R0133
dGTP 10mM	Thermo Scientific	cat# R0161
dCTP 10mM	Thermo Scientific	cat# R0151
DNA polymerase I 10U/ul	New England Bio Labs	cat# M0209L
RNaseH 10U/ul	Ambion	cat# AM2293
**End-Repair/A-Tailing and Adaptor Ligation**		
T4 DNA Ligase 5 Weiss U/µl +10x Buffer T4 DNA ligase	Thermo Scientific	cat# EL0011
T4 DNA polymerase 5U/µl	Thermo Scientific	cat# EP0062
T4 PNK 10U/ul	New England Bio Labs	cat# M0201L
Taq DNA polymerase 5U/ul	Thermo Scientific	cat# EP0402

10 mM dNTP	Thermo Scientific	cat# R0192
ATP 100mM	Thermo Scientific	cat# R0441
Adaptors. Included in Illumina TruSeq DNA LT Sample Prep Kit A and B	Illumina	FC-121-2001 and FC-121-2002
**Carboxylic acid (CA)-bead purification**		
Polyethylene glycol 8000 (PEG)	Sigma	cat# P1458-50ml
Dynabeads MyOne Carboxylic acid	Life Technologies (Invitrogen)	cat# 65012
Sodium chloride solution	Sigma	cat# 71386-1L
**Second strand Digestion with UDG**		
10x UDG Reaction Buffer	New England Bio Labs	cat# B0280S
UDG 5U/ul	New England Bio Labs	cat# M02805
**PCR Enrichment**		
PCR Primer Coctail. Included in Illumina TruSeq DNA LT Sample Prep Kit A and B	Illumina	cat# FC-121-2001 and FC-121-2002
2x Phusion Master Mix w. HF buffer	Thermo Scientific	cat# F-531L
**Other**		
Stratagene Universal Human Reference RNA	Agilent	cat# 740000
EB Buffer	Qiagen	Cat# 19086
