# Supplementary material for: The Sweden Cancerome Analysis Network - Breast (SCAN-B) Initiative: a large-scale multicenter infrastructure towards implementation of breast cancer genomic analyses in the clinical routine
Source: Genome Med. 2015 Feb 2;7(1):20. doi: 10.1186/s13073-015-0131-9 (PMC4341872; doi:10.1186/s13073-015-0131-9)
Supplement: Additional file 2: Figure S2. — Figure S3, Table S1, and Table S2. [file 13073_2015_131_MOESM2_ESM.pdf]

The Sweden Canceromics Analysis Network – Breast (SCAN-B) Initiative: a large-scale multicenter infrastructure towards implementation of breast cancer genomic analyses in the clinical routine

ADDITIONAL FILE 2

Lao H. Saal, Johan Vallon-Christersson, Jari Häkkinen, Cecilia Hegardt, Dorthe Grabau, Christof Winter, Christian Brueffer, Man-Hung Eric Tang, Christel Reuterswärd, Ralph Schulz, Anna Karlsson, Anna Ehinger, Janne Malina, Jonas Manjer, Martin Malmberg, Christer Larsson, Lisa Rydén, Niklas Loman, and Åke Borg

This appendix contains Figure S2, Figure S3, Table S1, and Table S2. The RNA-seq and microarray gene expression data with clinical and mutational annotations are available from the NCBI Gene Expression Omnibus under accession GSE60789.

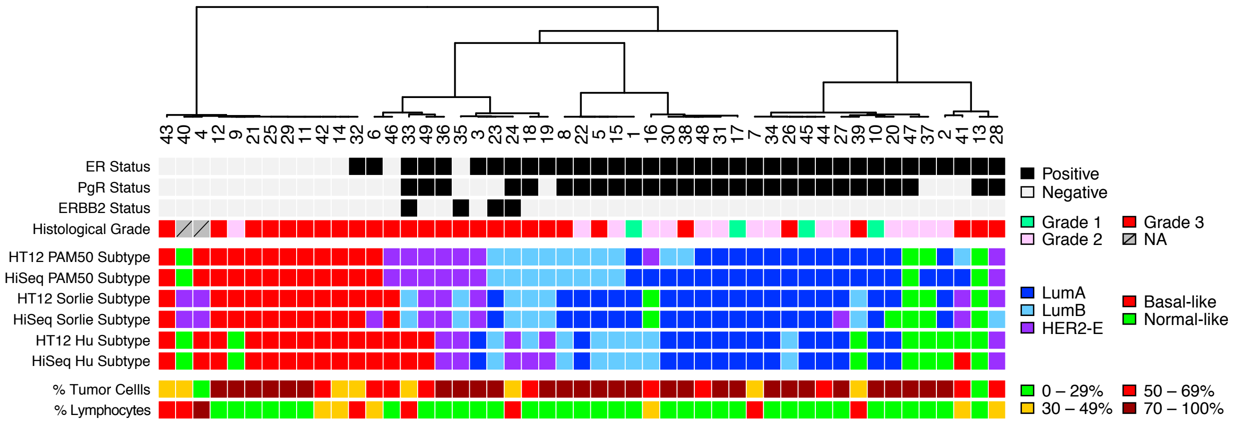

Figure S2 – Hierarchical clustering of 49 primary breast tumors using the RNA-seq gene expression measurements and the PAM50 intrinsic gene signature as in Figure 3.
Here, each tumor's molecular subtype is shown for three different signatures (PAM50, Sørlie, and Hu) using data from either RNA-seq (HiSeq) or microarray (HT12) platforms. See Materials and Methods section Molecular subtyping.

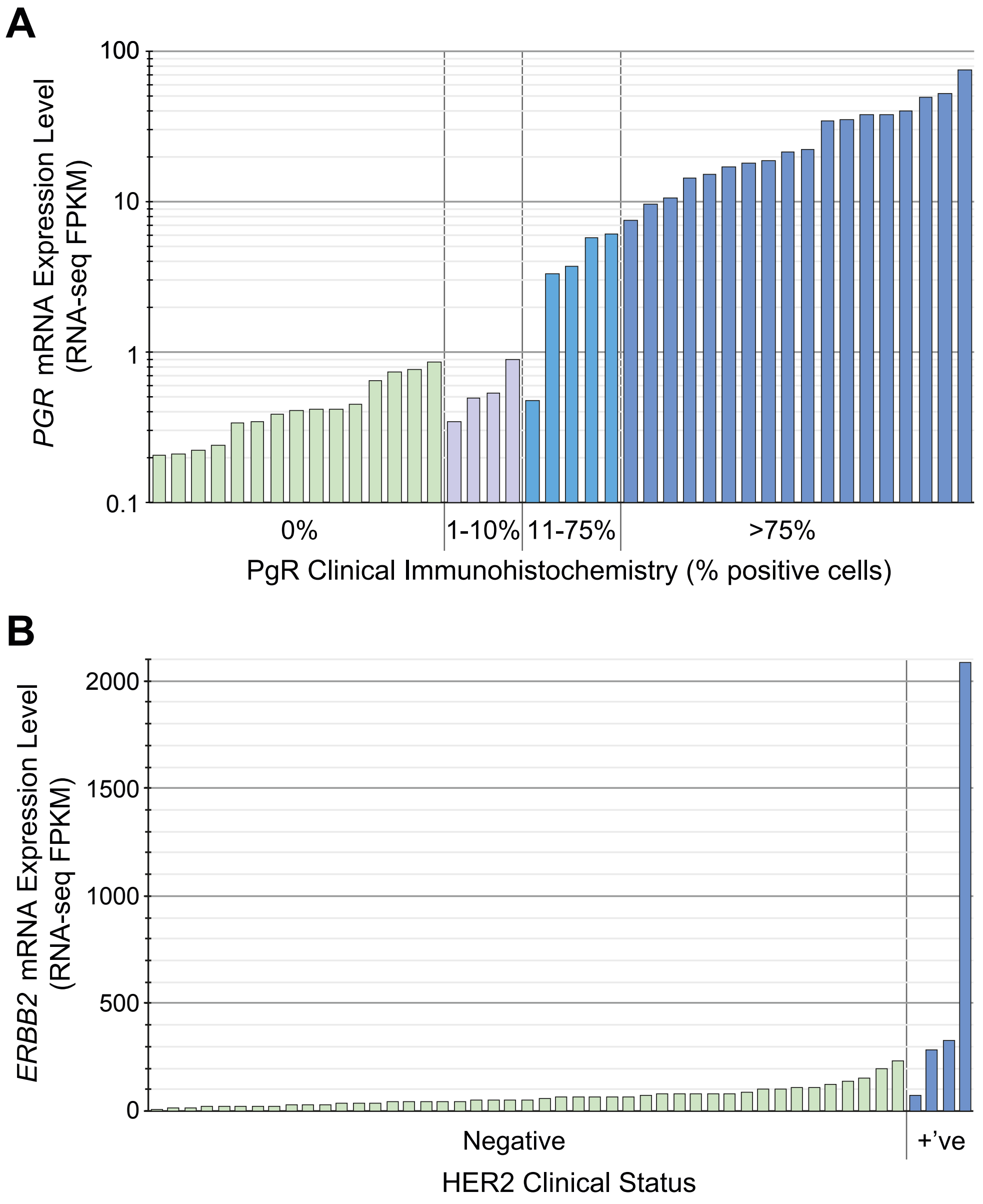

Figure S3 – RNA-seq-derived expression level of (A) *PGR*, which encodes the progesterone receptor (PgR), is shown compared to the clinical PgR IHC score for each tumor. Cases with missing percentage positive cells are not shown. In (B) the expression level of *ERBB2*, encoding the human epidermal growth factor receptor 2 (HER2), is shown compared to the clinical HER2 status.

Table S1. RNA-Seq Data

Tumor Number	Raw Sequencing Reads (million)	PF Rate	PF Reads (million)	PCF Rate	PCF Reads (million)	TopHat Alignment Rate	TopHat Aligned Reads (million)	Fraction PF Reads Mappable	Total Reads Aligned (million)	Fraction Unique Read-Pairs (non-duplicates)
1	74.9	82.5%	61.8	83.3%	51.5	68.9%	35.5	74.1%	45.8	70.2%
2	58.9	82.3%	48.5	81.9%	39.7	65.3%	26.0	71.6%	34.7	66.1%
3	54.3	89.3%	48.5	84.0%	40.8	72.3%	29.5	76.8%	37.2	69.8%
4	80.5	79.4%	63.9	87.2%	55.8	77.2%	43.0	80.1%	51.2	51.2%
5	53.9	81.5%	43.9	78.2%	34.3	70.6%	24.2	77.0%	33.8	74.6%
6	68.1	84.2%	57.3	76.8%	44.1	68.9%	30.4	76.1%	43.6	70.2%
7	21.0	89.6%	18.8	84.0%	15.8	61.9%	9.8	68.0%	12.8	24.9%
8	38.2	89.8%	34.3	80.3%	27.6	63.9%	17.6	71.0%	24.4	66.7%
9	48.4	89.5%	43.3	84.4%	36.6	67.4%	24.7	72.4%	31.4	58.3%
10	47.5	88.6%	42.1	84.1%	35.4	64.9%	22.9	70.5%	29.6	56.1%
11	53.2	84.9%	45.2	86.4%	39.0	76.0%	29.7	79.3%	35.8	54.9%
12	55.6	82.1%	45.7	87.8%	40.1	73.9%	29.7	77.1%	35.2	57.7%
13	68.2	82.1%	55.9	88.6%	49.6	65.3%	32.4	69.2%	38.7	66.2%
14	59.4	84.1%	49.9	84.2%	42.1	68.0%	28.6	73.0%	36.5	72.4%
15	47.5	84.3%	40.0	82.3%	33.0	61.8%	20.4	68.5%	27.4	40.4%
16	58.6	81.1%	47.5	85.6%	40.6	67.8%	27.5	72.4%	34.4	74.1%
17	71.3	83.4%	59.5	79.4%	47.2	71.0%	33.5	76.9%	45.8	68.3%
18	53.0	83.1%	44.0	82.2%	36.2	63.1%	22.8	69.6%	30.7	55.3%
19	61.2	83.6%	51.2	87.1%	44.6	76.9%	34.2	79.8%	40.9	51.1%
20	66.6	84.1%	56.0	86.6%	48.5	76.7%	37.2	79.8%	44.7	62.9%
21	56.6	83.4%	47.2	82.1%	38.7	78.1%	30.3	82.0%	38.7	53.9%
22	45.5	83.4%	38.0	87.3%	33.1	75.2%	24.9	78.3%	29.7	52.4%
23	48.8	83.5%	40.7	81.8%	33.3	61.6%	20.5	68.6%	27.9	47.9%
24	47.0	79.3%	37.2	79.5%	29.6	56.1%	16.6	65.1%	24.3	80.8%
25	59.8	89.7%	53.7	79.6%	42.7	65.4%	27.9	72.5%	38.9	55.4%
26	67.3	80.6%	54.3	84.7%	46.0	72.1%	33.1	76.4%	41.5	77.2%
27	69.2	83.8%	58.0	78.4%	45.5	75.5%	34.3	80.8%	46.8	65.8%
28	55.2	83.2%	45.9	82.2%	37.7	71.1%	26.8	76.3%	35.0	75.8%
29	60.5	82.6%	50.0	84.9%	42.4	64.1%	27.2	69.5%	34.7	43.6%
30	62.6	81.4%	50.9	86.5%	44.1	67.4%	29.7	71.8%	36.6	65.1%
31	49.5	84.4%	41.8	81.1%	33.9	64.6%	21.9	71.3%	29.8	44.2%
32	63.4	85.2%	54.1	76.1%	41.2	77.9%	32.1	83.2%	45.0	66.7%
33	55.9	84.6%	47.2	79.6%	37.6	76.6%	28.8	81.4%	38.5	63.3%
34	69.0	84.5%	58.3	85.7%	50.0	77.1%	38.5	80.4%	46.9	59.4%
35	75.8	82.0%	62.2	85.8%	53.4	65.4%	34.9	70.4%	43.8	65.8%
36	56.9	83.7%	47.6	73.0%	34.8	65.8%	22.9	75.1%	35.7	67.8%
37	55.8	81.9%	45.7	83.2%	38.0	67.5%	25.7	73.0%	33.3	70.9%
38	52.4	82.9%	43.4	84.9%	36.8	67.3%	24.8	72.3%	31.4	73.6%
39	67.4	84.5%	57.0	83.2%	47.4	65.3%	31.0	71.1%	40.5	61.8%
40	69.2	84.7%	58.6	86.4%	50.7	81.0%	41.0	83.6%	49.0	63.5%
41	42.1	86.0%	36.3	80.1%	29.1	76.3%	22.2	81.0%	29.4	44.6%
42	51.2	84.2%	43.1	79.9%	34.5	77.5%	26.7	82.0%	35.4	66.0%
43	65.7	80.3%	52.8	78.1%	41.2	73.6%	30.3	79.4%	41.9	59.3%
44	70.8	85.0%	60.1	84.7%	50.9	79.4%	40.4	82.6%	49.7	60.7%
45	58.2	83.3%	48.5	87.0%	42.2	67.7%	28.6	71.9%	34.9	72.8%
46	57.0	82.6%	47.1	80.9%	38.1	69.8%	26.6	75.6%	35.6	79.3%
47	51.6	82.9%	42.8	82.7%	35.4	61.6%	21.8	68.2%	29.2	55.5%
48	50.3	80.8%	40.6	83.8%	34.0	65.9%	22.4	71.4%	29.0	65.3%
49	56.9	83.5%	47.5	78.2%	37.2	68.0%	25.3	75.0%	35.6	53.0%
3-replicate	47.6	89.8%	42.7	82.6%	35.3	72.9%	25.7	77.6%	33.1	55.5%
10-replicate	50.1	89.3%	44.7	87.0%	38.9	63.0%	24.5	67.8%	30.3	47.7%
18-replicate	59.1	83.5%	49.3	81.6%	40.2	67.2%	27.0	73.2%	36.1	68.0%
22-replicate	70.8	82.7%	58.6	87.9%	51.5	77.1%	39.8	79.9%	46.8	59.2%
38-replicate	60.4	84.6%	51.1	84.4%	43.1	66.7%	28.8	71.9%	36.7	76.0%
45-replicate	64.0	84.2%	53.9	87.9%	47.4	62.9%	29.8	67.4%	36.3	50.1%
**Minimum**	21.0	79.3%	18.8	73.0%	15.8	56.1%	9.8	65.1%	12.8	24.9%
**Maximum**	80.5	89.8%	63.9	88.6%	55.8	81.0%	43.0	83.6%	51.2	80.8%
**Mean**	57.9	84.0%	48.5	83.0%	40.3	69.6%	28.2	74.7%	36.4	61.4%
**STDEV**	10.3	2.6%	8.2	3.4%	7.3	5.7%	6.3	4.8%	7.2	10.9%
**Median**	57.0	83.5%	47.6	83.3%	40.1	68.0%	27.9	74.1%	35.7	63.3%
**75th Percentile**	66.1	84.6%	54.2	85.7%	45.0	75.3%	31.5	79.3%	41.2	69.1%
**25th Percentile**	51.4	82.5%	43.4	80.6%	35.4	65.3%	24.6	71.2%	31.4	55.1%

Table S2. 90 Genes Screened for Mutations

Gene symbol	Gene name	Location	HGNC ID
AFF2	AF4/FMR2 family, member 2	Xq28	HGNC:3776
AKAP3	A kinase (PRKA) anchor protein 3	12p13.3	HGNC:373
AKT1	v-akt murine thymoma viral oncogene homolog 1	14q32.32-q32.33	HGNC:391
AKT2	v-akt murine thymoma viral oncogene homolog 2	19q13.1-q13.2	HGNC:392
APC	adenomatous polyposis coli	5q21-q22	HGNC:583
ARID1A	AT rich interactive domain 1A (SWI-like)	1p36.1-p35	HGNC:11110
ARID1B	AT rich interactive domain 1B (SWI1-like)	6q25.3	HGNC:18040
ARID2	AT rich interactive domain 2 (ARID, RFX-like)	12q13.11	HGNC:18037
ASXL1	additional sex combs like 1 (Drosophila)	20q11	HGNC:18318
ATM	ataxia telangiectasia mutated	11q22-q23	HGNC:795
ATN1	atrophin 1	12p	HGNC:3033
ATP2B2	ATPase, Ca++ transporting, plasma membrane 2	3p25.3	HGNC:815
BAP1	BRCA1 associated protein-1 (ubiquitin carboxy-terminal hydrolase)	3p21.31-p21.2	HGNC:950
BARD1	BRCA1 associated RING domain 1	2q34-q35	HGNC:952
BRCA1	breast cancer 1, early onset	17q21.31	HGNC:1100
BRCA2	breast cancer 2, early onset	13q12-q13	HGNC:1101
BRIP1	BRCA1 interacting protein C-terminal helicase 1	17q22.2	HGNC:20473
CASP8	caspase 8, apoptosis-related cysteine peptidase	2q33-q34	HGNC:1509
CBFB	core-binding factor, beta subunit	16q22.1	HGNC:1539
CCND1	cyclin D1	11q13	HGNC:1582
CCND3	cyclin D3	6p21	HGNC:1585
CDH1	cadherin 1, type 1, E-cadherin (epithelial)	16q22.1	HGNC:1748
CDKN1B	cyclin-dependent kinase inhibitor 1B (p27, Kip1)	12p13.1-p12	HGNC:1785
CDKN2A	cyclin-dependent kinase inhibitor 2A	9p21	HGNC:1787
CHEK2	checkpoint kinase 2	22q12.1	HGNC:16627
CLEC19A	C-type lectin domain family 19, member A	16p12.3	HGNC:34522
CTCF	CCCTC-binding factor (zinc finger protein)	16q21-q22.3	HGNC:13723
DCAF4L2	DDB1 and CUL4 associated factor 4-like 2	8q21.3	HGNC:26657
DGKG	diacylglycerol kinase, gamma 90kDa	3q27-q28	HGNC:2853
EP300	E1A binding protein p300	22q13.2	HGNC:3373
ERBB2	v-erb-b2 avian erythroblastic leukemia viral oncogene homolog 2	17q11.2-q12	HGNC:3430
ETV6	ets variant 6	12p13	HGNC:3495
FAM157B	family with sequence similarity 157, member B	9q34	HGNC:34080
FAM47C	family with sequence similarity 47, member C	Xp21.1	HGNC:25301
FOXA1	forkhead box A1	14q12-q13	HGNC:5021
GATA3	GATA binding protein 3	10p15	HGNC:4172
GPR32	G protein-coupled receptor 32	19q13.33	HGNC:4487
GPS2	G protein pathway suppressor 2	17p13.1	HGNC:4550
HIST1H1C	histone cluster 1, H1c	6p21.3	HGNC:4716
HIST1H2BC	histone cluster 1, H2bc	6p22.1	HGNC:4757
KCNB2	potassium voltage-gated channel, Shab-related subfamily, member 2	8q13.2	HGNC:6232
KRAS	Kirsten rat sarcoma viral oncogene homolog	12p12.1	HGNC:6407
MAP2K4	mitogen-activated protein kinase kinase 4	17p12	HGNC:6844
MAP3K1	mitogen-activated protein kinase kinase kinase 1, E3 ubiquitin protein ligase	5q11.2	HGNC:6848
MAP3K13	mitogen-activated protein kinase kinase kinase 13	3q27	HGNC:6852
MED23	mediator complex subunit 23	6q22.33-q24.1	HGNC:2372
MICA	MHC class I polypeptide-related sequence A	6p21.3	HGNC:7090
KMT2D	lysine (K)-specific methyltransferase 2D	12q13.12	HGNC:7133
KMT2C	lysine (K)-specific methyltransferase 2C	7q36	HGNC:13726
MRE11A	MRE11 meiotic recombination 11 homolog A (S. cerevisiae)	11q21	HGNC:7230
MYB	v-myb avian myeloblastosis viral oncogene homolog	6q22-q23	HGNC:7545
NCOR1	nuclear receptor corepressor 1	17p11.2	HGNC:7672
NF1	neurofibromin 1	17q11.2	HGNC:7765
NTRK3	neurotrophic tyrosine kinase, receptor, type 3	15q24-q25	HGNC:8033
OR2G3	olfactory receptor, family 2, subfamily G, member 3	1q44	HGNC:15008
OR2L2	olfactory receptor, family 2, subfamily L, member 2	1q44	HGNC:8266
OR6A2	olfactory receptor, family 6, subfamily A, member 2	11p15.4	HGNC:15301
PALB2	partner and localizer of BRCA2	16p12.1	HGNC:26144
PBRM1	polybromo 1	3p21	HGNC:30064
PIK3CA	phosphatidylinositol-4,5-bisphosphate 3-kinase, catalytic subunit alpha	3q26.3	HGNC:8975
PIK3R1	phosphoinositide-3-kinase, regulatory subunit 1 (alpha)	5q13.1	HGNC:8979
PIWIL1	piwi-like RNA-mediated gene silencing 1	12q24.33	HGNC:9007
PNPLA3	patatin-like phospholipase domain containing 3	22q13.31	HGNC:18590
PTEN	phosphatase and tensin homolog	10q23	HGNC:9588
PTPN22	protein tyrosine phosphatase, non-receptor type 22 (lymphoid)	1p13.2	HGNC:9652
PTPRD	protein tyrosine phosphatase, receptor type, D	9p24.1-p23	HGNC:9668
RAD50	RAD50 homolog (S. cerevisiae)	5q23-q31	HGNC:9816
RAD51C	RAD51 paralog C	17q25.1	HGNC:9820
RAD51D	RAD51 paralog D	17q11	HGNC:9823
RB1	retinoblastoma 1	13q14.2	HGNC:9884
RPGR	retinitis pigmentosa GTPase regulator	Xp11.4	HGNC:10295
RUNX1	runt-related transcription factor 1	21q22.3	HGNC:10471
RYR2	ryanodine receptor 2 (cardiac)	1q43	HGNC:10484
SEPT7P2	septin 7 pseudogene 2	7p12.3	HGNC:32339
SETD2	SET domain containing 2	3p21.31	HGNC:18420
SF3B1	splicing factor 3b, subunit 1, 155kDa	2q33.1	HGNC:10768
SMAD4	SMAD family member 4	18q21.1	HGNC:6770
SMARCD1	SWI/SNF related, matrix associated, actin dependent regulator of chromatin, subfamily d, member 1	12q13-q14	HGNC:11106
SRPR	signal recognition particle receptor (docking protein)	11q24-q25	HGNC:11307
STK11	serine/threonine kinase 11	19p13.3	HGNC:11389
TBL1XR1	transducin (beta)-like 1 X-linked receptor 1	3q26.33	HGNC:29529
TBX3	T-box 3	12q24.21	HGNC:11602
TLR4	toll-like receptor 4	9q33.1	HGNC:11850
TP53	tumor protein p53	17p13.1	HGNC:11998
TPRX1	tetra-peptide repeat homeobox 1	19q13.33	HGNC:32174
TRIM53AP	tripartite motif containing 53A, pseudogene	11q14.3	HGNC:19025
TRIM6-TRIM34	TRIM6-TRIM34 readthrough	11p15.4	HGNC:33440
USH2A	Usher syndrome 2A (autosomal recessive, mild)	1q41	HGNC:12601
WNT7A	wingless-type MMTV integration site family, member 7A	3p25	HGNC:12786
ZFP36L1	ZFP36 ring finger protein-like 1	14q22-q24	HGNC:1107
